# Hydroxyquinoline-coordinated organometallic complex nanowire and nanosheet for the dielectric layer of capacitors

**DOI:** 10.1039/d5na00450k

**Published:** 2025-07-28

**Authors:** Karim Khanmohammadi Chenab, Fardad Zarifi, Samaneh Mahmoudi Qashqay, Mohammad-Reza Zamani-Meymian

**Affiliations:** a Department of Chemistry, Iran University of Science and Technology Tehran P. O. Box 16846-13114 Iran karim.khanmohammadi90@gmail.com; b Department of Physics, Iran University of Science and Technology Tehran P. O. Box 16846-13114 Iran r_zamani@iust.ac.ir asamanehmahmoudi@gmail.com fardad939@gmail.com +98 21 7724 0497 +98 21 7322 5893

## Abstract

Understanding the mechanism of electron transfer in organometallic dielectric materials has been a major focus for capacitor applications. The present study reports four 8-hydroxyquinoline-based organometallic complexes and uses them as a dielectric layer for capacitors to analyze their capacitance (C), real and imaginary dielectric constants (*ε*′ and *ε*′′), loss factor (tan *δ*), dc and ac conductivity (*σ*_dc_ and *σ*_ac_), as well as the influence of morphology on their dielectric and electrical properties. These components were prepared using radio frequency (RF)-sputtering deposition and characterized by FESEM and LCRmetry methods. Molecular analysis of the dielectrics was undertaken using XRD, EDX, DRS, ^1^H and ^13^C NMR, Raman, FT-IR and PL spectroscopy techniques. Based on the results, an *f*-dependent damping of C was observed for AlQ_3_, ZnQ_2_ and CdQ_2_ dielectrics, while *ε*′ remained unchanged, and the *ε*′′ and tan *δ* of the dielectrics experienced a decrease and an increase *vs. f* and *T*, respectively. The value of *σ*_ac_ indicated an upward trend *vs. f*, which is linked to polarization of the nanowire and nanosheet dielectric layers. From the molecular aspect, symmetric structures inhibit aggregation of charge carriers and dipole contributions as well as interfacial polarization due to intramolecular charge transfer (ICT), metal-to-ligand charge transfer (MLCT) and ligand-to-metal charge transfer (LMCT) mechanisms. Finally, this research highlights the dielectric properties of organometallic materials to clarify the electron transfer (ET) mechanism for designing materials for dielectric layers.

## Abstract 2

Organometallic materials for application in dielectric layers of capacitors.

## Graphical abstract

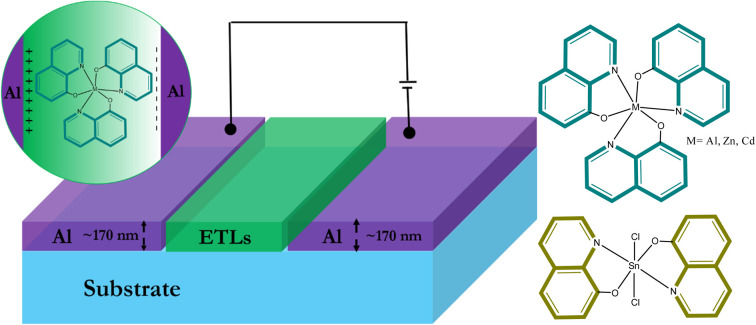

## 1. Introduction

In the Second Article of the Paris Climate Agreement, we read: “Holding the increase in the global average temperature to well below 2 °C above pre-industrial levels and pursuing efforts to limit the temperature increase to 1.5 °C above pre-industrial levels, recognizing that this would significantly reduce the risks and impacts of climate change”.^1^ This provides a landmark global vision and mission for developing sustainable and highly-efficient energy storage devices.^2^ Over the past decade, an understanding of the electron transfer (ET) mechanism in the dielectric layer of capacitors and the electron transfer layer (ETL) of organic light-emitting diodes (OLEDs) as sustainable energy storage devices has been the forefront of scientific discussion despite diligent efforts in this research area. In a capacitor, the dielectric layer serves as an insulating material between the conductive plates. While ideal dielectrics would store energy perfectly, real-world materials exhibit some energy dissipation, known as dielectric loss. This loss is manifested as heat generation within the dielectric material when an alternating current (ac) voltage is applied to the capacitor. A capacitor stores energy in the electric field, where the dielectric material is concentrated primarily between the conductive plates, and the charge polarization and separation are caused by dipole alignment, dipole reorientation and charge accumulation mechanisms. Despite general knowledge about the mechanism of action of the dielectric layer in capacitors, understanding of energy storage and transfer in organometallic dielectric materials is still in a state of ambiguity. In this regard, organometallic dielectric materials display some peerless advantages, such as higher dielectric constants than traditional inorganic dielectrics,^3,4^ low dielectric loss,^5^ responsiveness to external stimuli,^6^ and organic–inorganic hybridization. Together, these features open up possibilities for adaptive and reconfigurable devices as well as ultraflexible electronics applications.^7–9^ Capacitive deionization (CDI) of heavy metal ions from water sources is a technology that has emerged from within the properties of dielectric materials through effective adsorption and removal processes.^10^ Heteroatom-doped graphene–chitosan nanocomposites are one example of such materials developed by Qasemi *et al.*^11^ and Pang *et al.*^12^ that suggest bright horizons for future environmental applications. Cyclometalated organometallic complexes are the first generation of materials that have been utilized in multilayer OLED devices, due to their high external quantum efficiency (EQE) through both singlet and triplet excitons.^13–15^ Organometallic complexes are beneficial to ML_*n*_ molecular structures, permitting a change in the peripheral substitution of ligands around the central metal with electron-donating and electron-withdrawing substituents, in order to provide superior ability to tune the emission spectra from blue to red in OLEDs.^16,17^ For the first time, classical organometallic complexes have been applied as electroluminescent materials by introducing Ru(bpy)_3_^2+^, with wide photocatalytic applications in dye-sensitized solar cells (DSSCs).^18,19^ Among this plethora of complexes, in order to modify the redox potential of the excited state, alteration of the central metal as well as molecular engineering of the ligands have been the central paradigms of research to achieve the greatest matching of Fermi levels in electrode design. Thus, tris(8-hydroxyquinolinato)aluminum, known as AlQ_3_, has been a milestone in emitting ETMs in OLED technologies.^20^ More recently, by using organometallic semiconductors and AlQ_3_, efficient spin-polarized injection and transport were obtained in spintronic devices in the organic–inorganic interface channels of AlQ_3_.^21^ Based on the fact that these organometallic semiconductors have been applied for designing vertical spin valve-based devices with an AlQ_3_ layer and a bottom manganite La_0.7_Sr_0.3_MnO_3_ (LSMO) electrode, the presence of a direct interface between the AlQ_3_ layer and the top insulating tunnel barrier under the cobalt electrode make them appropriate not only for standard spintronic applications but also for fabricating new OLEDs and organic field-effect transistors (OFETs).^22^ It is well known that spin–orbit coupling (SOC) is responsible for the induced dephasing of coherent spins through transport in normal semiconductors. To solve this challenge, by applying the integrating capability of organometallic materials in hybrid organic–inorganic interfaces, low SOC perturbations were obtained by developing sexithiophene as the organic channel material in a hybrid with spin-polarized manganite LSMO-based electrodes.^23^ This means that the AlQ_3_ complex provided a low-temperature giant magneto-resistance effect, which led to the pioneering work of Tang and Van Slyke in developing highly efficient AlQ_3_-based OLEDs. In this regard, there have been waves of research to explore the fluorescence and phosphorescence properties of AlQ_3_-based organometallic semiconductors.^24–27^ The main goal of this research is to calculate the dielectric properties of organometallic complexes based on the optical properties of AlQ_3_, ZnQ_2_, CdQ_2_ and SnQ_2_Cl_2_, which open up a fundamental understanding of ETL materials for capacitor applications. At the same time, through this study, analysis of physicochemical properties of these materials was carried out by determining the capacitance values, real and imaginary dielectric constants, dc and ac conductivity, as well as the impact of molecular structures on the loss factors of the relevant capacitors. On the one hand, the present study concentrates on the characterization of these organometallic materials using spectroscopy and microscopy analysis. On the other hand, the influence of MLCT and LMCT on the rate of ET in such organometallic dielectric materials was investigated by considering various frequencies and temperatures, disclosing the importance of such calculations.

## 2. Experimental

The synthesis routes for tris(8-hydroxyquinoline)aluminum(iii) (AlQ_3_), bis(8-hydroxyquinoline)cadmium(ii) (CdQ_2_), bisdichloro(8-hydroxyquinoline)tin(ii) (SnQ_2_Cl_2_), and bis(8-hydroxyquinoline)zinc(ii) (ZnQ_2_), and the fabrication of the capacitor devices follow previously reported methods.^28–30^ The key commercial grade materials that have been utilized in this paper are 8-hydroxyquinoline (8-HQ, 98.5% Sigma-Aldrich Co.), aluminum nitrate nonahydrate (Al(NO_3_)_3_·9H_2_O, 98.5%, Merck Co.), cadmium nitrate tetrahydrate, Cd(NO_3_)_2_.4H_2_O (99.0%, Merck Co.), tin(ii) chloride dihydrate (SnCl_2_·2H_2_O, 99.99%, Merck Co.), zinc nitrate hexahydrate (Zn(NO_3_)_2_.6H_2_O, 99.0%, Sigma-Aldrich Co.), ethanol (99.9%, Merck Co.), methanol (99.9%, Merck Co.), *N*,*N*-dimethylformamide (DMF, 99.8%, Merck Co.), dimethyl sulfoxide (DMSO, 99.9%, Merck Co.), acetone (99.9%, Merck Co.), and KOH (85%, Merck Co.).

### 2.1. Synthesis of AlQ_3_

The synthesis of AlQ_3_ was initiated by dissolving 8-HQ (1.40 g) in 10 mL of ethanol under stirring until a transparent orange solution of 8-HQ was obtained. Aluminum nitrate nonahydrate (3.0 g in 40 mL) aqueous solution was added to the mixture. Then, an aqueous solution of KOH (0.858 g in 12 mL) was added dropwise into the mixture to increase nucleophilic substitution and complete the reaction. Then, this new mixture and the aluminum nitrate nonahydrate mixture were added together, and a suspension of olive-colored product was obtained after stirring for 20 min. The final product was washed several times using ethanol and DI-water and recrystallized in ethanol, and dried at 40 °C for 1 h. Thus, a pure AlQ_3_ complex was obtained.

### 2.2. Synthesis of CdQ_2_

In a similar procedure, 2.90 g of 8-HQ was dissolved in 15 mL of methanol, and then, it was stirred thoroughly until an orange transparent solution was obtained. Then, 3.6 g of cadmium nitrate tetrahydrate was separately dissolved in 7.0 mL of methanol and stirred gently until a transparent crystal solution was obtained. Then, both mixtures were combined, and an aqueous solution of KOH (0.858 g in 12 mL) was added dropwise into the mixture, and the resultant suspension was stirred for 20 min. The final product was filtered using filter papers and then dried at room temperature, and thus a pure CdQ_2_ complex was obtained.

### 2.3. Synthesis of SnQ_2_Cl_2_

To synthesize SnQ_2_Cl_2_, 2.15 g of 8-HQ was dissolved in 15 mL of methanol, and then, the mixture was stirred until an orange transparent solution was obtained. Then, using 4.3 g of tin(ii) chloride dihydrate, 25 mL of methanol solution was prepared, and both mixtures were combined. An aqueous solution of KOH (0.858 g in 12 mL) was added dropwise into the mixture until a suspension of the product was obtained after stirring for 20 min. The final product was dried at room temperature on filter papers and thus a pure complex of SnQ_2_Cl_2_ was obtained.

### 2.4. Synthesis of ZnQ_2_

Preparation of the ZnQ_2_ complex was started by dissolving 3.41 g of 8-HQ in 20 mL of methanol under stirring until an orange transparent solution was obtained. Simultaneously, 3.5 g of zinc nitrate hexahydrate was dissolved in 20 mL of methanol and was added to the first solution. Then, 1.668 g of KOH dissolved in 15 mL of water was added to initiate the reaction. A suspension of the product was obtained by adding KOH solution dropwise after stirring for 20 min. The final product was dried at room temperature for 45 min after filtering and washing as well as recrystallizing with pure methanol and DI-water.

### 2.5. Purification procedure

By applying the recrystallization method, AlQ_3_, ZnQ_2_, CdQ_2_ and SnQ_2_Cl_2_ were purified as much as possible in ethanol to remove unreacted 8-HQ ligands and inorganic salts. The precipitates were extensively double-washed with DI-water and methanol. The formation of nanowire and nanosheet morphologies ratified the pure synthesis reaction conditions and dropwise addition of reactants. For synthesis of the dielectrics, ethanolic solutions of 8-HQ and Al(NO_3_)_3_·9H_2_O, Cd(NO_3_)_2_, SnCl_2_·2H_2_O and Zn(NO_3_)_2_·6H_2_O were utilized, while the pH of the reaction medium was kept in the 11–12 range with KOH solution. The reactions provided yellow and green precipitates that easily accumulated at the bottom of the reaction flux. The pH of the precipitates was neutralized by washing several times with ethanol and DI-water until the pH was fixed at 7, and the precipitates were dried under vacuum. Therefore, purification of the precipitates was achieved in three main steps: removal of unreacted 8-HQ, pH neutralization to remove hydroxide and metal ions, and the removal of moisture in a vacuum oven.

### 2.6. Electrode preparation

Fabrication of electrodes for the capacitor device was implemented by previously reported sputtering methods.^31–33^ RF-sputtering instruments provide advantages, such as uniform thickness and coatings with a dense structure and a high deposition rate, and they were utilized previously for thin layer deposition of ZrO_2_ and TaN and In_2_O_3_.^34,35^ The fabrication process of the electrodes was started by thin film deposition of Al using an rf-sputtering device (Desk Sputter Coater DST3, Nano-Structured Coatings Co., Iran) on glass substrates, which had been prepared with dimensions of 1.0 × 1.0 × 0.2 cm. Before this step, the glass samples were treated by washing in ethanol and acetone under ultrasonic conditions for 20 min at room temperature (RT). Then, the instrument chamber with a pure Al target (Sindlhauser Materials GmbH; purity: 99.99%) was evacuated using a turbopump (Pfeiffer Vacuum GmbH) to reach a pressure of about 1.96 × 10^−5^ Torr, while the flow rate of Ar gas was kept at 33 atm cm^3^ min^−1^, known as standard cubic centimeter per minute (sccm), using a mass flow controller (MFC, Line Tech Co.), which supplied a pressure of about 2.86 × 10^−2^ Torr. The deposition process started with a pre-sputtering step for 10 min using a 100 W power supply, while the thickness of the deposited layer was detected by a quartz crystal system in the instrument.

### 2.7. Thin layer deposition of dielectrics

The deposition of 8-hydroxyquinoline-based organometallic complex dielectrics was implemented using a sol ink method, in which the inks were formulated by dispersing 0.1 g of AlQ_3_, CdQ_2_, SnQ_2_Cl_2_ and ZnQ_2_ dielectrics separately in 1.0 mL of DMF for five minutes under ultrasonic conditions at RT. Then, the sol inks were deposited between the electrodes in Al-based substrates using a spin-coating method^36^ and were dried at RT for 24 h. In addition, copper wire back contacts were attached to the electrodes using Ag glue for measurement.

## 3. Characterization

### 3.1. Molecular structures of dielectrics

The Fourier transform-infrared (FT-IR) spectroscopy of the dielectrics was conducted to analyze the molecular structures of AlQ_3_, CdQ_2_, SnQ_2_Cl_2_, and ZnQ_2_ powders using an FT-IR spectrometer (PerkinElmer, Inc., USA), which provides the transmission *vs.* wavenumber for the samples.^37^ The FT-IR spectroscopy method was initiated by making KBr tablets for the dielectrics and pressing them under a pressure of about 10 tonne cm^−2^. Using ^1^H and ^13^C-NMR (500 MHz, Bruker Co., USA) devices, the molecular structures of the dielectrics were investigated by gently dispersing pure dielectric powder (0.003 g) in DMSO and CDCl_3_ NMR solvents, through the green-tape NMR tubes method.^38^ Additionally, the crystalline structures of the dielectrics were determined by applying an X-ray diffraction (XRD) spectrometer (D8 advance, Bruker Co., USA), and data in the 2*θ* range from 5 to 95° were collected at RT for AlQ_3_, CdQ_2_, SnQ_2_Cl_2_, and ZnQ_2_ organometallics using a D/max 2400 X-ray diffractometer tools equipped with Cu Kα radiation (*λ* = 1.54050 Å).^39^

### 3.2. Spectroscopy

Diffuse reflectance spectroscopy (DRS) analysis for 8-HQ organometallics was performed using a DRS apparatus (2550, Shimadzu Co. Japan) to consider the maximum wavelength (*λ*_max_) *vs.* absorption profile. Absorption profile analysis of the dielectrics was implemented using powdered samples. Additionally, photoluminescence spectroscopy (PL) of 8-HQ organometallics was implemented using a fluorescence spectrophotometer with a 355 nm wavelength laser for excitation (Varian Cary Eclipse, Agilent Co. USA) to consider the intensity and wavelength of emissions from 8-HQ organometallics.^40^ Energy-dispersive X-ray spectroscopy (EDX) using an SEM instrument (VEGA3 XMU, TESCAN Co., Czech Republic) was used to identify the elemental composition of the materials. DRS, Shimadzu Co.) of the ETMs was implemented to measure the band gap (*E*_g_) of the dielectrics based on eqn (1):^41^

1 (*αhν*)^2^ = *A*(*hν* − *E*_g_)

where *A*, *hν*, *α* and *E*_g_ represent the proportionality constant, incident light frequency, absorption coefficient and band gap, respectively. Photoluminescence (PL) spectroscopy was performed on powdered samples in a fluorescence spectrometer (PerkinElmer, Inc., *λ*_ex_ = 355 nm) equipped with a monochromatized xenon-lamp-based excitation source. A Raman microspectrophotometer (ram-532–004, DPSS Nd: YAG laser, *λ* = 532 nm and *P* = 200 mW, spectral resolution = 0.7 nm, Technooran Co., Iran) was applied for Raman spectroscopy of the organometallic dielectrics.

### 3.3. LCRmetry

Analysis of the capacitance of the capacitors was conducted in the frequency range from 10^2^ to 10^4^ Hz at various temperatures in the range *T* = 35–60 °C. The value of tangent loss (tan *δ*) for the capacitors was analyzed using a portable LCRmeter (ESCORT ELC-133A model, Taiwan). In this regard, to derive the dielectric constant, ac conductivity and other relevant parameters, eqn (2)–(4) were utilized, as follows:^42–48^2
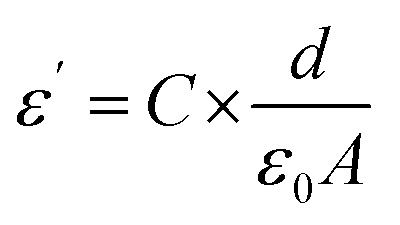

3 *σ*_ac_ = 2π*fε*_0_*ε*′ tan *δ*

4
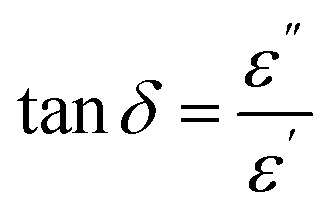
where *δ*, *σ*_ac_, *ε*_0_, *ε*′′, *ε*′, *f*, *A*, *d* and *C* represent the loss factor of the capacitors, ac conductivity (Ω^−1^ m^−1^), the permittivity of free space (8.854 × 10^−12^ F m^−1^), the imaginary dielectric constant, the real dielectric constant, applied frequency (Hz), the area of the electrodes (*m*^2^), thickness (*m*) and capacitance (*F*), respectively.

### 3.4. Interfacial characterization

The morphology and thickness of the electrodes were characterized using a field-emission scanning electron microscope (FESEM, Zeiss). An Au sputtering process was implemented in a vacuum chamber (EHT, 10 kV) before surface composition analysis in EDS mode (Zeiss Co. Germany, Oxford Instruments Co. UK). An X-ray diffraction (XRD) spectroscope (Panalytical Co. UK) was applied to determine thin-layer crystallinity. The Debye–Scherrer equation (eqn (5)) was applied as follows:^49^5
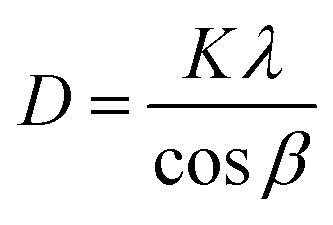
where *D*, *K*, *λ* and *β* represent the crystalline size of the nanoparticles, the Scherrer constant, which is about 0.98, wavelength (1.54050 Å) and the full width at half maximum (FWHM). The interplanar spacings of the orthorhombic, monoclinic and triclinic crystalline unit cell dimensions were calculated using eqn (6)–(8), respectively, as follows:^50,51^6
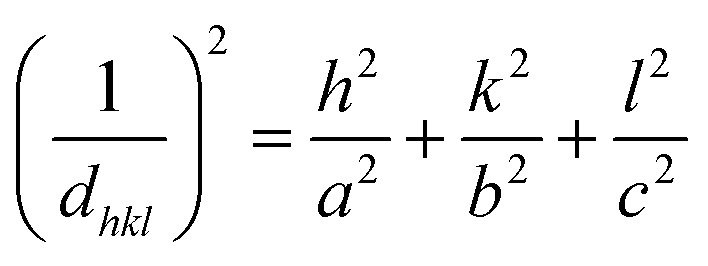
7
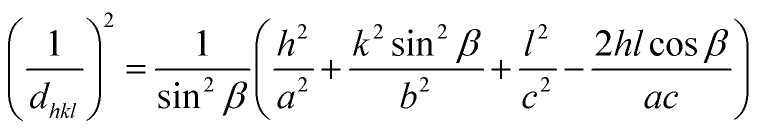
8
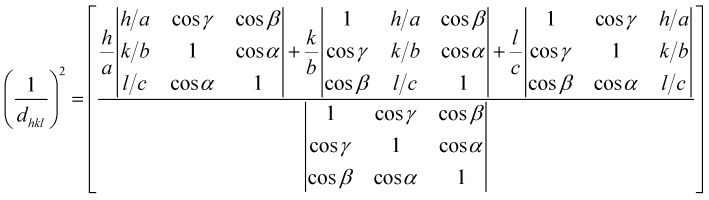
where *hkl*, *a*, *b*, *c*, *α*, *β* and *γ* represent Laue indices and crystal unit cell dimensions, respectively.

## 4. Results and discussion

### 4.1. Characterization of organometallic dielectrics

**Fig. 1:**
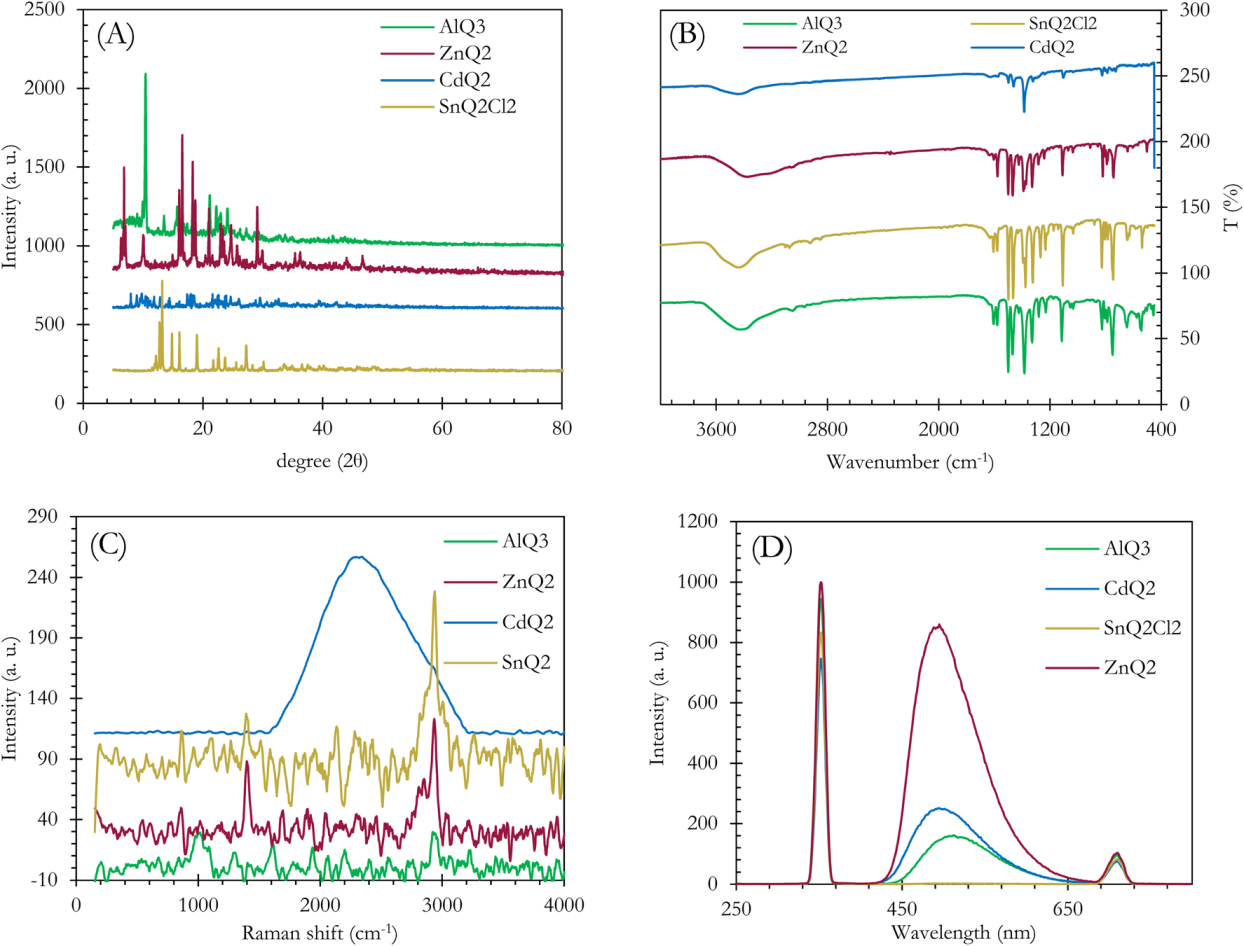
(A) XRD spectra of AlQ_3_, CdQ_2_, SnQ_2_Cl_2_ and ZnQ_2_ organometallic complexes. (B) FT-IR spectra of AlQ_3_, CdQ_2_, SnQ_2_Cl_2_ and ZnQ_2_ organometallic complexes. (C) Raman shifts for AlQ_3_, CdQ_2_, SnQ_2_Cl_2_ and ZnQ_2_. (D) PL analysis.

**Fig. 2:**
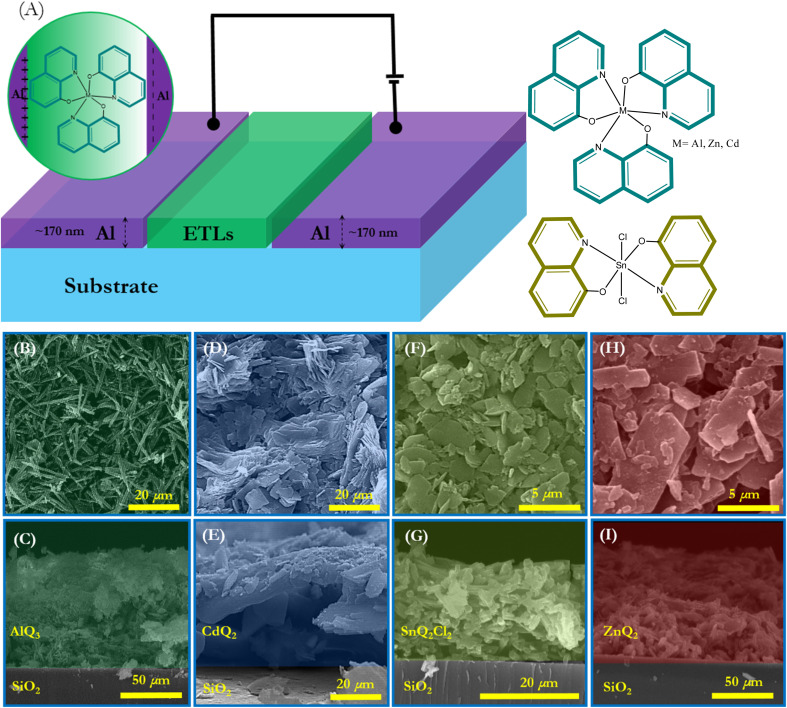
(A) Schematic illustration of capacitors with dielectric layers of AlQ_3_, ZnQ_2_, CdQ_2_ and SnQ_2_Cl_2_, respectively, and their organometallic molecular structures. (B–I) FESEM and cross-sectional microscopy of AlQ_3_, ZnQ_2_, CdQ_2_ and SnQ_2_Cl_2_, respectively, on glass substrates.

**Fig. 3:**
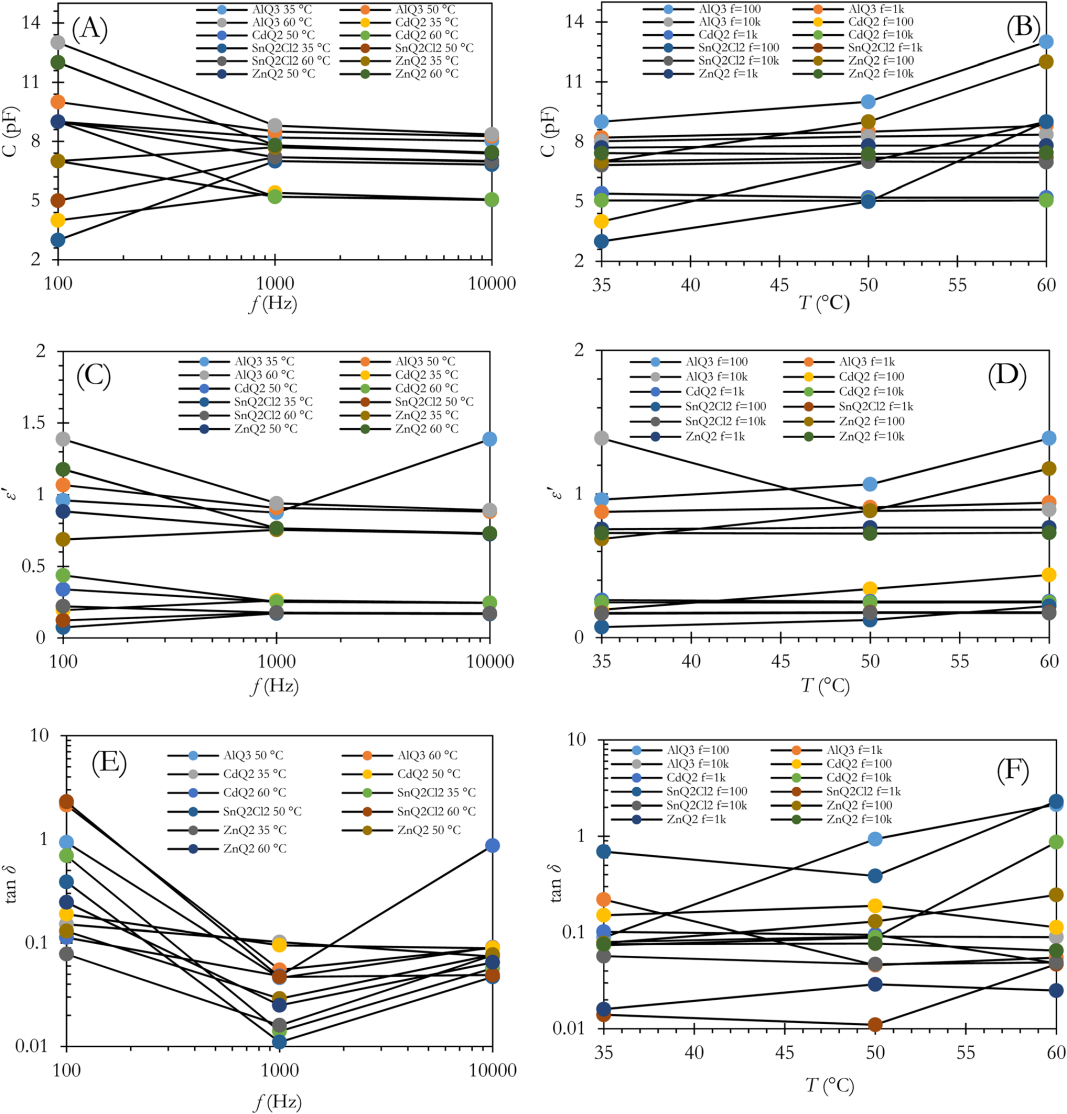
The *C*, tan *δ* and *ε*′ of capacitors with AlQ_3_, ZnQ_2_, CdQ_2_ and SnQ_2_Cl_2_ dielectrics *vs. T* and *f*. (A and B) The alteration of *C vs. f* and *T*. (C and D) Profiles of *ε*′ *vs. f* and *T*. (E and F) The tan *δ* *vs. f* and *T*.

**Fig. 4:**
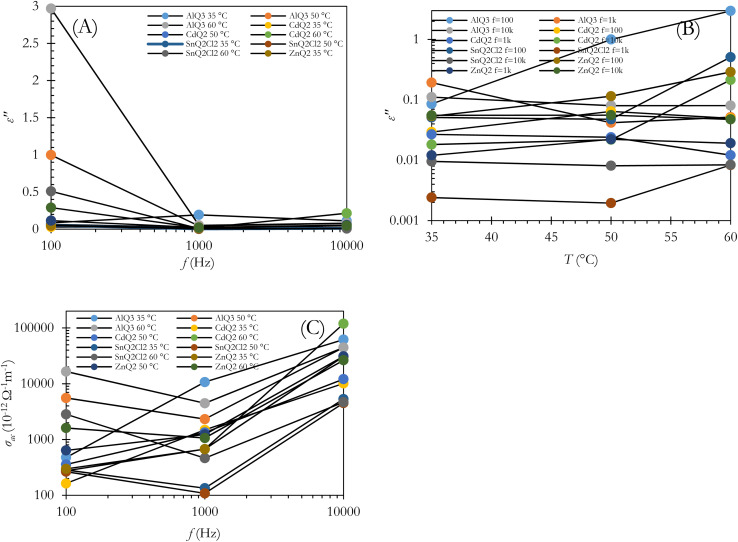
Profiles of *ε*′′ and ac conductivity (*σ*_ac_) for capacitors with AlQ_3_, ZnQ_2_, CdQ_2_ and SnQ_2_Cl_2_ organometallic dielectric layers *vs. f* (A and C) and *vs. T*(B).

The AlQ_3_, ZnQ_2_, CdQ_2_ and SnQ_2_Cl_2_ organometallic electron transfer materials (ETMs) were synthesized through a rapid complexation reaction. Initially, powdered AlQ_3_, ZnQ_2_, CdQ_2_ and SnQ_2_Cl_2_ were analyzed to identify their crystalline structures using X-ray diffraction (XRD) spectroscopy. Based on the results (Fig. 1A), AlQ_3_, ZnQ_2_, CdQ_2_ and SnQ_2_Cl_2_ provided standard patterns of diffraction, demonstrating the effective method of synthesis of the ETM dielectrics. Based on the XRD patterns of the ETMs, AlQ_3_, ZnQ_2_, and CdQ_2_ were found to be consistent with JCPDS reference codes 26-1550, 48-2116 and 96-700-3504. This confirmed that AlQ_3_ belongs to the triclinic crystal system, with space group *P*1̄, with two isomeric forms of AlQ_3_, called “facial” and “meridional”, with unit cell dimension values of about *a* = 13.58 Å, *b* = 12.44 Å, and *c* = 7.75 Å, and *α* = 69.90°, *β* = 89.47°, and *γ* = 82.52°, respectively. SnQ_2_Cl_2_ belonged to an orthorhombic crystal system with space group *Pbca* and unit cell dimension values of about *a* = 16.3675 Å, *b* = 13.7121 Å and *c* = 17.4519 Å, with *α* = *β* = *γ* = 90°, and a cell volume of 3916.8 Å^3^. While ZnQ_2_ had a space group of *P*1̄ with unit cell dimensions of *a* = 10.8537 Å, *b* = 11.8814 Å, and *c* = 13.0532 Å, and *α* = 74.119°, *β* = 73.449°, and *γ* = 70.999°, and volume = 1494.9 Å^3^. Finally, CdQ_2_ belonged to the monoclinic crystal system with *C*12/*c*1 space group and lattice dimensions of about *a* = 25.0980 Å, *b* = 10.7350 Å and *c* = 17.7430 Å, with *α* = *β* = 90°, *γ* = 128.7070°, calculated density = 1.83 g cm^−3^, plus cell volume of 3730.44 × 10^6^ pm^3^. For AlQ_3_, groups of peaks at 2*θ* of 9.947, 10.411, 13.501, 15.691, 16.803, 18.438, 21.141, 22.237, 22.945, 24.089 and 27.251° represented Laue indices (*hkl*) of (010), (01−1), (002), (020), (01−2), (02–1), (023), (1–1–2), (132), (−103) and (1–3–2), with interplanar spacing values (*d*_hkl_) of about 8.8851, 8.4900, 6.5534, 5.6432, 5.2721, 4.8080, 14.1991, 3.9945, 3.8729, 3.6914 and 3.2698 Å, respectively. For SnQ_2_Cl_2_, the results were 12.157, 12.748, 13.200, 14.830, 16.078, 18.995, 21.731, 22.608, 23.695, 25.547, 28.293, 30.132, 33.573 and 36.557° with *d*_hkl_ of 7.2741, 6.9386, 6.7019, 5.9689, 5.5083, 4.6684, 4.0863, 3.9297, 3.7520, 3.4839, 3.2717, 3.1518, 2.9634 and 2.4560 Å, respectively. While for ZnQ_2_, they were determined at 2*θ* of 6.306, 10.037, 16.044, 16.549, 18.293, 18.734, 21.015, 23.299 and 29.076° for *hkl* of (200), (023), (111), (034), (121), (052), (112), (002) and (332), with *d*_hkl_ of 14.0052, 12.6691, 8.8050, 5.5198, 5.3523, 4.8459, 4.7328, 4.2240, 3.8148, 306 019 and 3.0684 Å, respectively. Finally, for CdQ_2_, the results for 2*θ* were 7.986, 10.509, 14.343, 18.183, 21.620, 24.625 and 29.554° for *hkl* of (200), (111), (401), (302), (402), (203) and (413), with *d*_hkl_ of 11.0616, 8.4115, 6.1701, 4.8749, 4.1071, 3.6121 and 3.0201 Å, respectively. The XRD pattern of the AlQ_3_ complex shows polymorphism in the crystalline structure, while in SnQ_2_Cl_2_, two Cl ligands can coordinate to the Sn central metal and form a *cis* isomeric structure for this organometallic complex, which was confirmed by EDX analysis (Fig. 3S, SI). ZnQ_2_ and CdQ_2_ can make pseudo-dimeric crystalline structures that can play an impactful role in their electronic and physicochemical structures. The element composition of the AlQ_3_, ZnQ_2_, CdQ_2_ and SnQ_2_Cl_2_ organometallic ETMs was confirmed by performing EDX analysis (Fig. 1–4S, SI). Based on the results, C, N, and O atoms of the 8-HQ ligand were confirmed for ETMs, as well as the presence of Al, Zn, Cd, Sn and Cl atoms. For AlQ_3_, K*α*_C_, K*α*_N_, K*α*_O_, K*α*_Al_, K*α*_K_ and K*β*_K_ were 0.25, 0.3, 0.55, 1.5, 3.3 and 3.6 keV, respectively (Fig. 4S, SI). Similarly, for ZnQ_2_, K*α*_C_, K*α*_N_, K*α*_O_, K*α*_Zn_, K*β*_Zn_, L*α*_Zn_, K*α*_K_ and K*β*_K_ were 0.25, 0.3, 0.55, 8.7, 9.6, 1.0, 3.3 and 3.6 keV, respectively (Fig. 2S, SI). Finally, for CdQ_2_, K*α*_C_, K*α*_O_, L*α*_Cd_, L*β*_Cd_, K*α*_K_ and K*β*_K_ were 0.25, 0.55, 3.2, 3.3, 3.3 and 3.6 keV, and for SnQ_2_Cl_2_, K*α*_C_, K*α*_N_, K*α*_O_, L*α*_Sn_, L*β*_Sn_, K*α*_Cl_ and K*β*_Cl_ were 0.25, 0.3, 0.55, 3.4, 3.7, 2.7 and 2.8 keV, respectively (Fig. 1S and 3S, SI). EDX analysis confirmed the presence of Cl atoms in SnQ_2_Cl_2_ samples. The remaining and unreacted KOH from the synthesis process is the main reason for the appearance of K atoms in these analyses (Fig. 1S, SI). The molecular structures of the AlQ_3_, ZnQ_2_, CdQ_2_ and SnQ_2_Cl_2_ organometallic ETMs were initially investigated using FT-IR spectroscopy (Fig. 1B), which demonstrated the characteristic peaks of 8-HQ ligand structures and the central metal ion–ligand bonds. For example, the characteristic bands at *ν* 600–800 cm^−1^ relate to vibrations of 8-HQ ligands and the fingerprint peaks at *ν* 400–600 cm^−1^ are attributed to metal (M: Al^3+^, Zn^2+^, Cd^2+^ and Sn^2+^)–ligand stretching vibration. Additionally, the intense and weakened peaks at *ν* 1251 and 1280.6 cm^−1^ relate to C–O functional groups (FGs), confirming the formation of an M–O bond in AlQ_3_, ZnQ_2_, CdQ_2_ and SnQ_2_Cl_2_ organometallic structures. The vibrations of ring-stretching and C–H bending were revealed in lines at *ν* 1329, 1380, and 1499 cm^−1^,^52,53^ while the peak at *ν* 3055 cm^−1^ relates to the C–H FG stretching vibration in the 8-HQ ligand aromatic ring. The stretching vibrations of the –OH FGs appeared as a wide peak at *ν* 3417 cm^−1^, while the stretching modes of C–N FGs could be found at *ν* 1390 and 1328 cm^−1^. The *ν* at 3417 cm^−1^ confirmed the coordination nature of the M–O, (M: Al^3+^, Zn^2+^, Cd^2+^ and Sn^2+^) bonds, which are not ionic in nature.^54^ As the characteristic and prominent peaks of the aromatic ring skeleton of 8-HQ that appear at *ν* 1588, 1550, 1467 and 1400 cm^−1^ rely on the impact of the intense conjugation effect in aromatic rings, as well as all hybridization of π-electron bonding orbitals. The decrease in intensity and the disappearance of peaks at *ν* 460 and 710 cm^−1^ confirm the presence of the coupling effect on vibrational modes between Al^3+^, Zn^2+^, Cd^2+^ and Sn^2+^ ions and ligands. The Raman activity and polarizability of the AlQ_3_, ZnQ_2_, CdQ_2_ and SnQ_2_Cl_2_ organometallic ETMs were investigated by Raman spectroscopy. This technique provides useful information about the ground-state and excited-state energy of the organometallic compounds as well as the MLCT mechanism, with an emphasis on the vibrational transitions of the central metal and ligands. Respectively, for AlQ_3_, ZnQ_2_, CdQ_2_ and SnQ_2_Cl_2_ organometallics, the characteristic Stock (*ν*_s_) and anti-Stoke (*ν*_as_) peaks as well as Rayleigh scattering (*ν*_r_), can be observed as Raman shifts in Fig. 1C. The vibrational bands at *ν* 1595 and 1394 cm^−1^ relate to the *ν*_ring_ and [*ν*_ring_ + *δ*(C–H)] of 8-HQ, while other lower-frequency combined peaks appeared at *ν*_ring_ 757 cm^−1^, [*ν*(Al–O) + *ν*(Al–N) + *δ*_ring_)] at *ν*_ring_ 647 and 541 cm^−1^, [*δ*_ring_ + *ν*(Al–O)] at *ν*_ring_ 525 cm^−1^, and *δ*_ring_ at *ν*_ring_ 504 cm^−1^. Also, the modes of [*ν*(C–N) + *δ*(C–H)] and [*ν*_ring_ + *ν*(C–O) + *δ*(C–H)] appeared at 1230 and 1335 cm^−1^, respectively. PL spectroscopy provides useful information about the effects of intermolecular interactions and packing on optical properties. The PL of AlQ_3_, CdQ_2_ and ZnQ_2_ organometallics (Fig. 1D) appeared at *λ*_max,emission_ of 515, 498, 491 nm, for green emission, while for SnQ_2_Cl_2_, the Cl^−^ ligands play a quenching role and inhibit fluorescence properties. These peaks can be attributed to several factors, including: (1) the presence of intramolecular charge transfer (ICT) – in particular, MLCT from Al^3+^, Cd^2+^ and Zn^2+^ to the 8-HQ ligands–causes radiative relaxation between S_1_ and S_0_ states. (2) referring to Kasha's rule, intense nonradiative internal conversion occurs between vibrational S_2_ and S_1_ excited states and also between S_1_ and S_0_ states, which could be related to LLCT among 8-HQ ligands through π → π* electronic transitions. (3) The presence of a small peak at 714 nm, which relates to S_1_ → S_0_ radiative emission from semi-stable vibrational states to S_0_. (4) The strong intersystem crossing (ISC) of the organometallics between S_1_ and T_1_ can occur through spin–orbit coupling (SOC) of the d_π_ orbitals of Al, Cd, Sn and Zn with the ππ* orbitals of 8-HQ, providing strong phosphorescence properties through T_1_ → S_0_ transitions, as well as delayed radiative fluorescence emission. The corresponding S_1_ → T_1_ rate, *k*_ISC_, through ISC can be expressed by eqn (9):9
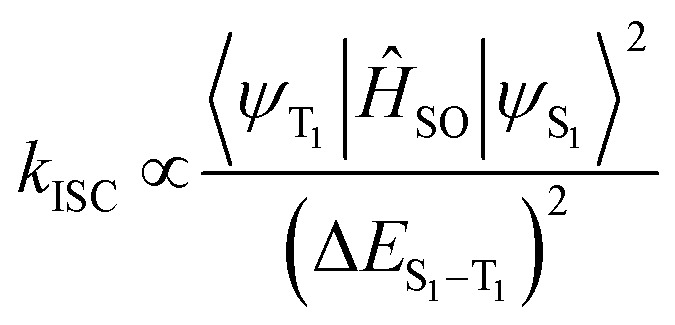
where *Ĥ*_SO_ and Δ*E*_S_1_−T_1__ represent the Hamiltonian operator of SOC and the energy difference between S_1_ and T_1_ states, while the mixing of ππ* orbitals with MLCT between Al, Cd, Sn and Zn and 8-HQ in both S_1_ and T_1_ states causes an S_1_ → T_1_ ISC process in the incorporated forms of 〈^1^d_π_π*|*Ĥ*_SO_|^3^ππ*〉 or 〈^3^d_π_π*|*Ĥ*_SO_|^1^ππ*〉 terms. In addition, the T_1_→S_0_ radiative transition rate *k*_r_^p^ can be written as eqn (10)–(12):10
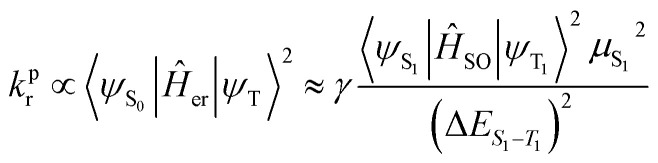
11
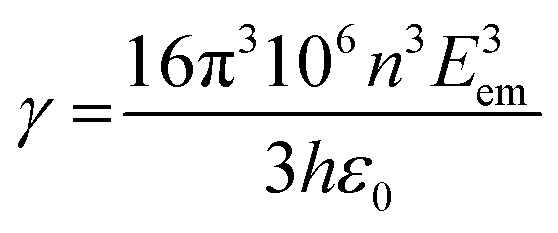
12
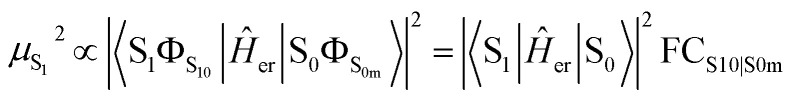
where *Ĥ*_er_, *E*_em_, *n*, *ε*_0_, *h* and *μ*_S_1__ represent the electric dipole operator, the T_1_ → S_0_ energy gap, the refractive index, permittivity in a vacuum, Planck's constant and the S_0_ → S_1_ transition dipole moment, respectively. FC_S10,S0m_ defines the Franck–Condon overlap factor, which occurs in the S_1_ vibrational wavefunction (*Φ*) at *ν* = 0 and in S_0_ at *ν* = *m*. ^1^H-NMR provides useful information about the number of protons and an understanding about the chemical environment of each of them in the molecular structure. The formation of new bonds and any change in its environment could be tracked by the absence or shift of a particular proton. One important blueprint in this regard is the absence of a proton peak related to –OH FGs on 8-HQ, which is attributed to the formation of coordination bonds between Al, Cd, Sn and Zn atoms (Fig. 2S, SI). Basically, the proton peak of the –OH FG of 8-HQ appears at around 10 ppm, but for none of the AlQ_3_, ZnQ_2_, CdQ_2_ or SnQ_2_Cl_2_ organometallics was such a peak especially observed, but some peaks were found around 6.6 to 6.9 ppm as well as 8.4 to 8.8 ppm. These findings were also confirmed and supported by FTIR and Raman spectroscopy data. As could be observed from the NMR profiles, the protons in the –N–CH– peak appeared at 8.8 ppm while a peak at 8.2 ppm could be assigned to –N–CH–CH–CH– protons. Two aromatic protons of 8-HQ ligands appeared at 7.4–7.6 ppm, while the protons of –O–C–CH– were found at 7.0 ppm (Fig. 6–9S, SI).

### 4.2. Capacitance (C) analysis

As shown in Fig. 2A, an illustration of the dielectric organometallic complexes was applied to develop capacitors with an Al|dielectric|Al structure. In this design, AlQ_3_, CdQ_2_, SnQ_2_Cl_2_ and ZnQ_2_ were used as dielectric thin films in the sandwiched architecture of the capacitor, while measurement of the dielectric constant (*ε*′) for AlQ_3_, CdQ_2_, SnQ_2_Cl_2_ and ZnQ_2_ organometallic complexes was possible by investigating the capacitance (C) values. It is well known that *ε*′ can play a crucial role in disclosing the concepts of intramolecular charge transfer (ICT) and polarizability of the organometallic complexes, especially the metal centers. The electron-donating or electron-withdrawing ability and oxidation state of the metal center can have a significant impact on ICT. With reference to the significant influence of ionic radius and charge on the electronegativity of Al^3+^, Cd^2+^, Sn^2+^ and Zn^2+^ ions, they can be ordered as Al^3+^ > Cd^2+^ > Zn^2+^ > Sn^2+^. This trend consequently leads to the following order of ICT strength: AlQ_3_ > CdQ_2_ > ZnQ_2_ > SnQ_2_Cl_2_. It should be noted that the MLCT and LMCT mechanisms can occur in the molecular structure of these organometallic complexes. Moreover, as the presence of metal atoms often enhances polarizability, thereby increasing dielectric response under an external electric field for these dielectric materials, considering electron–hole mobility and charge carriers to reveal the mechanism of ET can be regarded as the conceptual novelty of this research. The electron–hole mobility (*μ*_h_–_e_) in the AlQ_3_, CdQ_2_, SnQ_2_Cl_2_ and ZnQ_2_ dielectric layer is related to *ε*′, based on the space-charge-limited current (SCLC) model in Mott–Gurney theory following eqn (13):^55,56^13
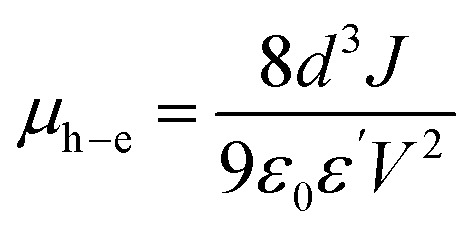
where *V*, *J*, *d* and *μ*_h–e_ represent the potential of the device, current density, the thickness of the dielectric layer and electron–hole mobility, respectively. In the Al|dielectric|Al architecture of the capacitor, the thickness of dielectric layers (d), which also provides the distance between the Ag electrodes, could be obtained from cross-FESEM of the capacitors. Fig. 2B–I provide a cross-sectional FESEM image of the capacitor and thickness of each thin layer, which for the dielectric layers provided *d* values of about 94.52, 86.8, 43.03 and 21.78 μm for AlQ_3_, ZnQ_2_, CdQ_2_ and SnQ_2_Cl_2_, respectively. Using the LCRmetry method, the C profiles for AlQ_3_, ZnQ_2_, CdQ_2_ and SnQ_2_Cl_2_ were measured at various temperature to investigate its impacts (Fig. 2A and B).

As shown in Fig. 3A and B, an *f*-dependent decrease in C was observed for the AlQ_3_, ZnQ_2_ and CdQ_2_ dielectrics from 10^2^ to 10^4^ Hz at 35, 50 and 65 °C. For the AlQ_3_-based capacitor, the C values were 9.0, 8.2 and 8.02 pF for 10^2^, 10^3^ and 10^4^ Hz at *T* = 35 °C, respectively. The results for *T* = 50 and 65 °C were obtained as 10.0, 8.5 and 8.26 pF, and 13.0, 8.8 and 8.35 pF, respectively. For AlQ_3_, *ε*′ was measured as 0.96, 0.87 and 1.38 for 10^2^, 10^3^ and 10^4^ Hz at *T* = 35 °C, respectively (Fig. 2C and D). Under the same conditions, the *C* values of ZnQ_2_ were recorded as 7.0, 7.7 and 7.47 pF, and *ε*′ were 0.68, 0.75, 0.73, respectively. Accordingly, the corresponding results for the CdQ_2_ dielectric were obtained as 4.0, 5.4 and 5.06 pF, and 0.19, 0.26 and 0.24 for *ε*′, respectively. Diverse results were obtained for the SnQ_2_Cl_2_ dielectric at 35 and 50 °C. For example, the C values were obtained as 3.0, 7.0 and 6.82 pF at 35 °C with an increasing profile. A similar result was observed for 0.07, 0.16 and 0.17 for *ε*′ for 10^2^, 10^3^ and 10^4^ Hz, respectively. Such a descent in the *C* values of the dielectrics *vs. f* can be assigned to tan *δ* as loss factors for the capacitors (Fig. 3E and F). Mathematically, tan *δ* can be defined as tan *δ* = *ε*′′/*ε*′, in which *ε*′′ represents the imaginary dielectric constant.^57,58^ Also, based on the complex dielectric permittivity (*ε*) equation, *ε* = *ε*′ + *iε*′′,^59^ tan *δ* describes the amount of energy loss in capacitors in charge mode. Based on this fact, the value of the change in C *vs. f* in the Goswami and Goswami model can be formulated as a relation between equivalent series of capacitance (*C*_S_) and *f* using eqn (14):^60^14
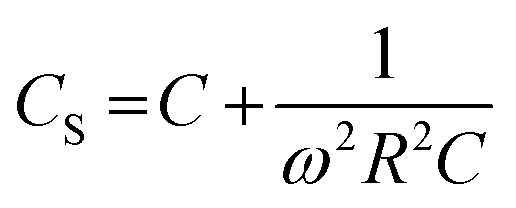
where *R* represents the discrete resistance element and *ω* = 2π*f*. Based on Fig. 3, the *C* of the capacitors undergoes an enhancement *vs. T* that relates directly to greater charge carrier accumulation at higher *T*. Such results have previously been reported for dye molecules like phthalocyanines^61^ and triphenylamines.^62,63^ Penn's model^64^ provides a direct relation between the band gap (*E*_g_) of the dielectric and the *ε*′ value, as in eqn (15) and (16):^65^15
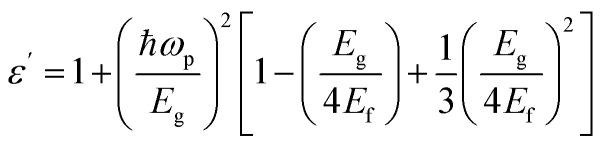
16
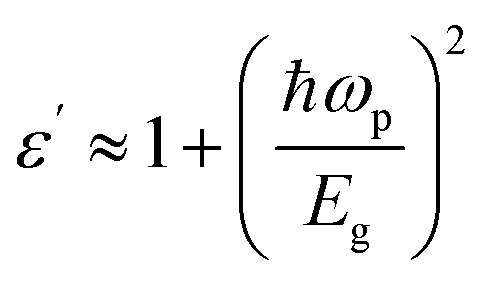
where in these equations the plasma frequency, *ω*_p_^2^, could be defined as *ω*_p_^2^ = 4π*N*e^2^/*m.* Additionally, ℏ, *m*, *E*_f_ and *N* represent Plank's constant, electron effective mass, Fermi energy and electron concentration. For a small value of *E*_g_/4*E*_f_, *ε*′ ∝ 1/*E*_g_^2^, and a small alteration in the *E*_g_ value can have a striking influence on *ε*′. Additionally, it is expected that the *E*_g_ of AlQ_3_, ZnQ_2_, CdQ_2_ and SnQ_2_Cl_2_ dielectrics will depend on their organometallic structures, as well as central metals and ICT that will potentially have an impact on *ε*′. At a frequency of 10^2^ Hz and also in higher-frequency domains, *ε*′ did not exhibit any substantial change in value (Fig. 3). AlQ_3_, ZnQ_2_ and CdQ_2_ with *C*_3v_ and SnQ_2_Cl_2_ with *D*_2h_ point groups are beneficial for symmetric molecular structures, inhibiting accumulation of charge carriers and significant contributions of dipoles as well as interfacial polarization.

### 4.3. Loss factor (tan *δ*) of organometallic dielectrics

As *ε* can be written as *ε* = *ε*′ *+ iε*′′,^59^ tan *δ* could be formulated as tan *δ* = *ε*′′/*ε*′,^57,58^ which physically describes the value of energy loss in capacitors. As an unwanted structural shortcoming of a capacitor, energy loss can occur for several physical reasons, including: (1) a polarization shift in grain boundaries behind the electric field;^66,67^ (2) migration of interfacial polarization space charges; (3) inducing dc conductivity in some spot points of the dielectric layer; and (4) the Stark effect, which can potentially be observed in the molecular dipoles.^68^ It should be noted that the Stark effect can be formulated as Δ*f* = −Δ*μE* − 1/2*E*Δ*αE*, where Δ*α*, *E*, Δ*μ* and Δ*f* represent the change in polarizability of the organometallic molecule, the local electric field, the change in dipole moment and the transition in frequency. Fig. 4A and B indicate the *ε*′′ and tan *δ* of the dielectrics *vs. f* at *T* = 35, 50 and 65 °C. The damping of the results can be interpreted as interfacial polarization of the dielectrics in the capacitor. From the morphological point of the view, as shown in the cross-sectional and top-view FESEM images of the dielectric layers in Fig. 2B–I, the porosity of the dielectric layers as well as the morphology of the microstructures for AlQ_3_, ZnQ_2_, CdQ_2_ and SnQ_2_Cl_2_ were one-dimensional wires and two-dimensional sheets that can decrease tan *δ* by reducing dc conductivity. Also, the agglomeration morphology on the Al conductive substrate electrode confirmed a dense morphology. Such a wire-like morphology of AlQ_3_ and also the sheet-like microstructures of ZnQ_2_, CdQ_2_ and SnQ_2_Cl_2_ can be attributed to molecular interactions between them. The top-view FESEM images (Fig. 2B –I) indicate that the grain size for AlQ_3_ differs not only in shape but also in size, exhibiting smaller grains compared to ZnQ_2_, CdQ_2_ and SnQ_2_Cl_2_. Such a difference in size influences charge diffusion dynamics in the dielectric thin layer.^69^ A slight increase in the ac conductivity of the dielectrics in the high-frequency domain corresponds to fast polarization in dielectric layers (Fig. 4C).^70^ According to the theory of Gurevich and Tagantsev, intrinsic dielectric loss can be affected by symmetry in the crystal structure of the dielectric layer, ac field frequency, and the layered morphology of a dielectric composite,^67,71^ as well as temperature.^72^ Based on Fig. 3E and F, the alteration profile of tan *δ* for AlQ_3_ is more intense than those for ZnQ_2_, CdQ_2_ or SnQ_2_Cl_2_ and is proportional to *T* (tan *δ* ∝ *T*), while unchanged profiles *vs. T* were obtained for ZnQ_2_, CdQ_2_ and SnQ_2_Cl_2_ that can be attributed to the two-phonon process of dielectric loss in the AlQ_3_ dielectric layer. Such diversities could arise not only from the crystalline structures of each dielectric but also from their micro-grain morphology.

## 5. Conclusion

This study focused on designing four organometallic complexes, AlQ_3_, CdQ_2_, SnQ_2_Cl_2_ and ZnQ_2_, as electron transfer materials (ETMs) and considering their dielectric properties for application in the dielectric layer of capacitors. Using these dielectric materials in Al|dielectric|Al architectured capacitors, the characteristic parameters were determined by analyzing capacitance (C), real and imaginary dielectric constants (*ε*′and *ε*′′), loss factor (tan *δ*), and dc and ac conductivity (*σ*_dc_ and *σ*_ac_). Then, the impact of the morphology of the dielectric layers was investigated by designing components of capacitors by rf-sputtering deposition and spin-coating methods. Field emission scanning electron microscopy (FESEM) and LCRmetry were applied to analyze characteristics at diverse temperatures (*T*) from 35 to 50 °C and frequencies from 10^2^ to 10^4^ Hz. Additionally, microscopic analysis of organometallic dielectrics in top-view and cross-sectional modes indicated nanowire and nanosheet-like morphologies for these materials. The results of LCRmetry revealed an *f*-dependent decreasing profile for C for the AlQ_3_, ZnQ_2_ and CdQ_2_ dielectrics from 10^2^ to 10^4^ Hz at 35, 50 and 65 °C, while the *ε*′ values experienced an unchanged profile. For *ε*′′ and tan *δ* of the dielectrics, decreasing and increasing values *vs. f* and *T* were obtained, respectively. The *σ*_ac_ as the ac conductivity of the dielectrics provided an upward trend *vs. f*, which was attributed to the fast polarization in the dielectric layers due to their wire and sheet-like morphologies. Symmetrical molecular structures of organometallic dielectrics inhibit the aggregation of charge carriers and significant contributions of dipoles, as well as interfacial polarization. Such properties that arise from intramolecular charge transfer (ICT), as well as metal-to-ligand charge transfer (MLCT) and ligand-to-metal charge transfer (LMCT), can open up new prospects for designing new dielectric materials for capacitors.

## Conflicts of interest

No conflicts to declare.

## Supplementary Material

NA-OLF-D5NA00450K-s001

## Data Availability

The data supporting this article including EDX analysis of dielectrics, DRS analysis of dielectrics, ^1^H- & ^13^C NMR analysis of dielectrics. See DOI: https://doi.org/10.1039/d5na00450k.
