# Supplementary material for: Hydroxyquinoline-coordinated organometallic complex nanowire and nanosheet for the dielectric layer of capacitors
Source: Nanoscale Adv. 2025 Jul 28;7(19):6098–109. doi: 10.1039/d5na00450k (PMC12366522; doi:10.1039/d5na00450k)
Supplement: NA-007-D5NA00450K-s001 [file NA-007-D5NA00450K-s001.pdf]

Supplementary Information (SI) for Nanoscale Advances.
This journal is © The Royal Society of Chemistry 2025

Hydroxyquinoline-coordinated Organometallic Complexes Nanowire and Nanosheet for Dielectric Layer of Capacitors

Karim Khanmohammadi Chenab[1,2], Fardad Zarifi[2], Samaneh Mahmoudi Qashqay[2], Mohammad-Reza Zamani-Meymian[2*]

[1] Department of Chemistry, Iran University of Science and Technology, P.O. Box 16846-13114, Tehran, Iran.

[2] Department of Physics, Iran University of Science and Technology, P.O. Box 16846-13114, Tehran, Iran.

Corresponding Author: Mohammad-Reza Zamani-Meymian*, Email: r_zamani@iust.ac.ir, Telephone Number: (+98) 21 7322 5893. Fax: (+98) 21 7724 0497

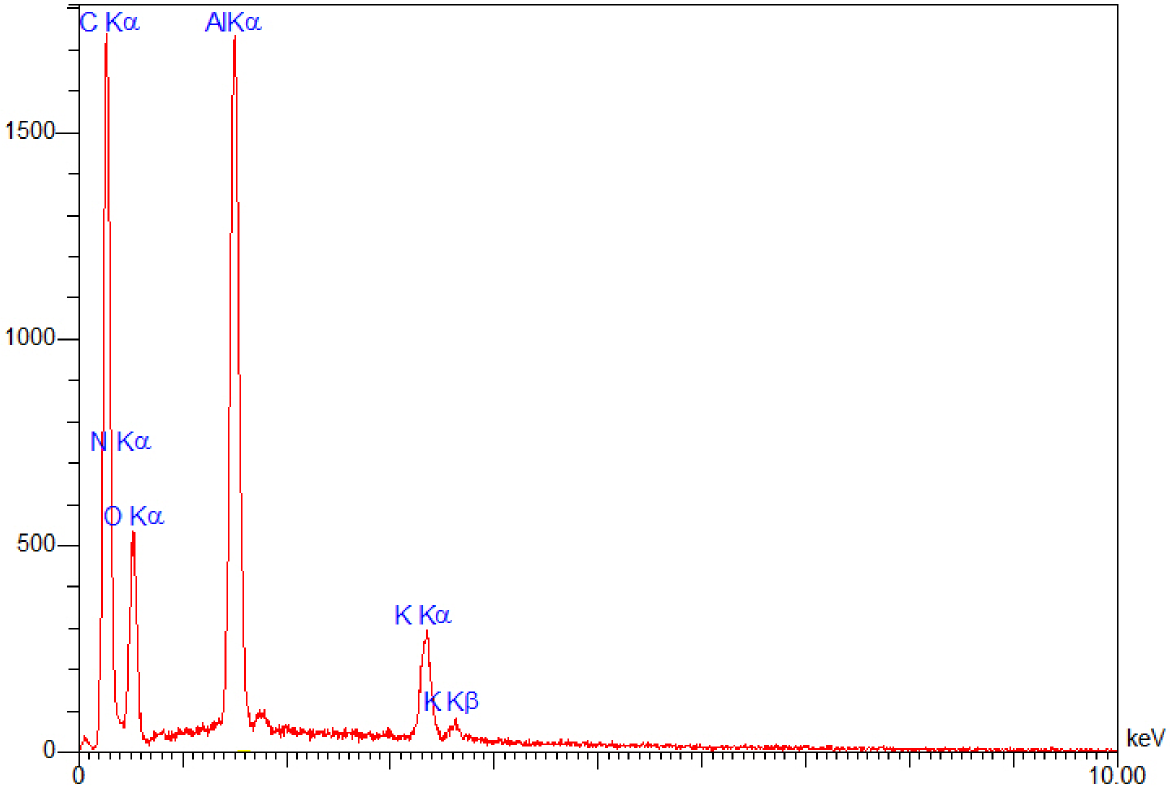

Figure 1S. EDX analysis of AlQ_3.

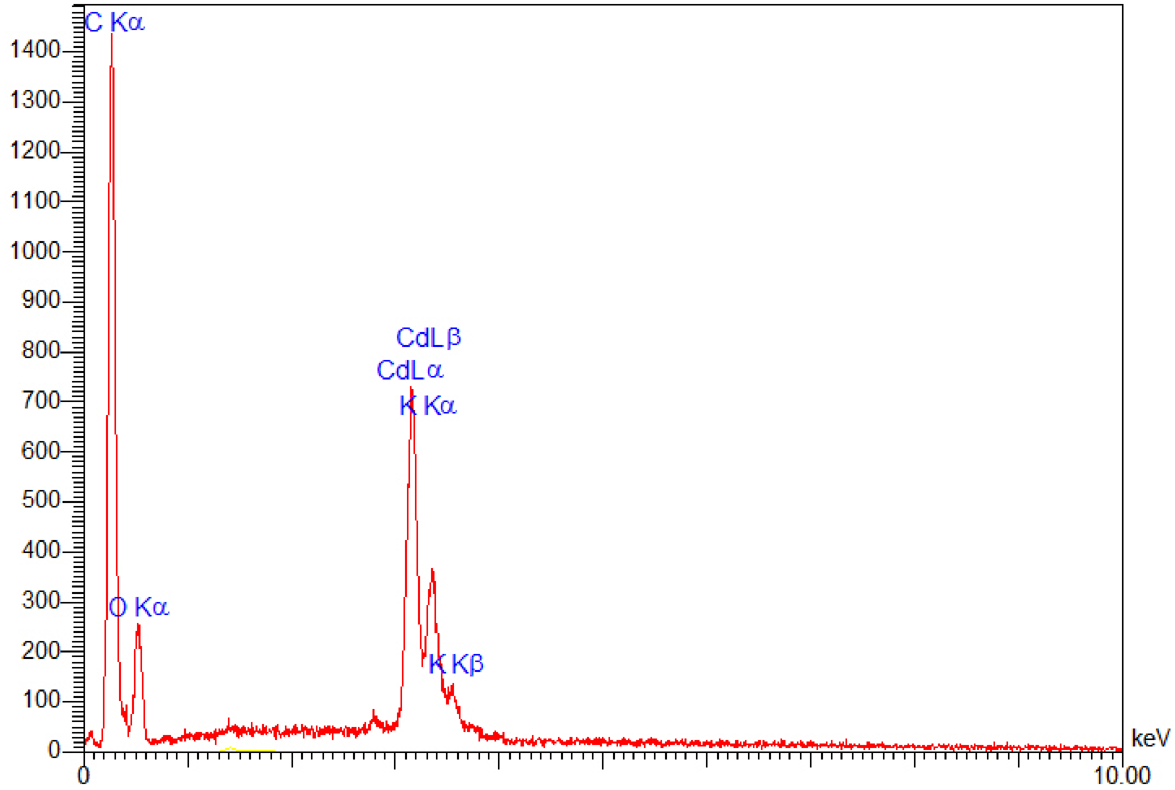

Figure 2S. EDX analysis of CdQ_2**.**

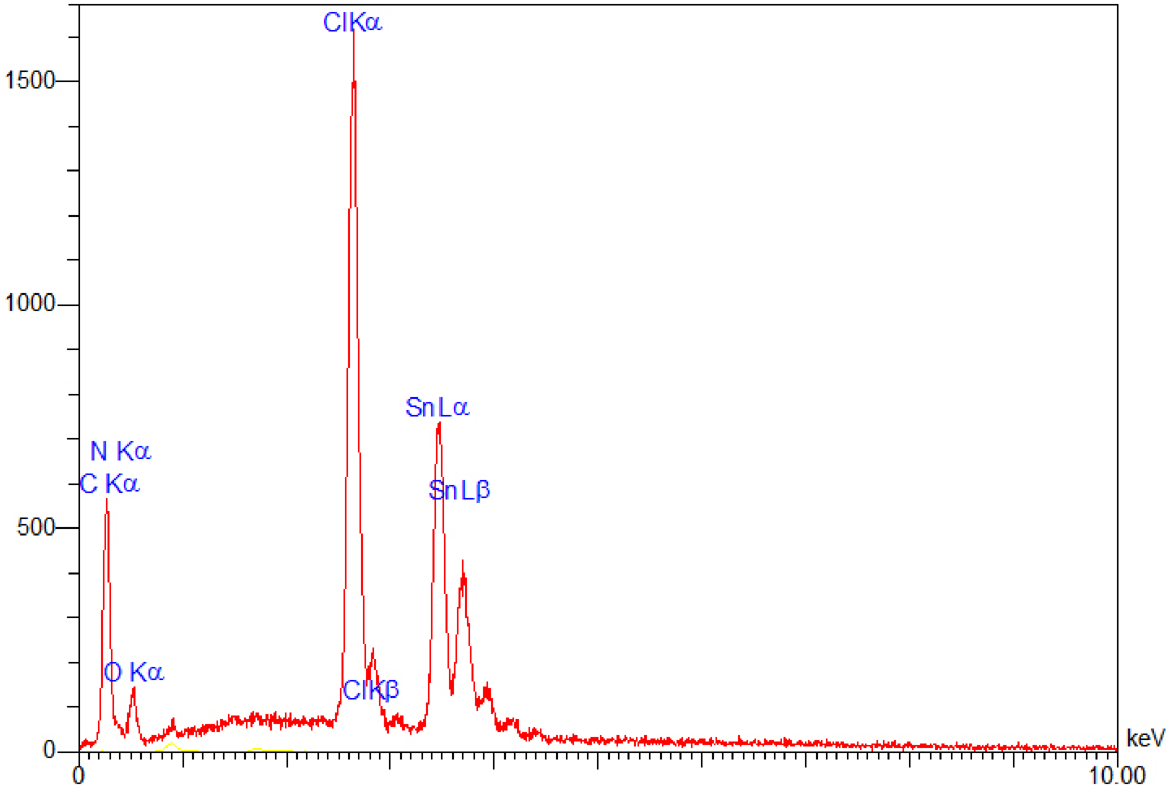

Figure 3S. EDX analysis of SnQ_2Cl_2.

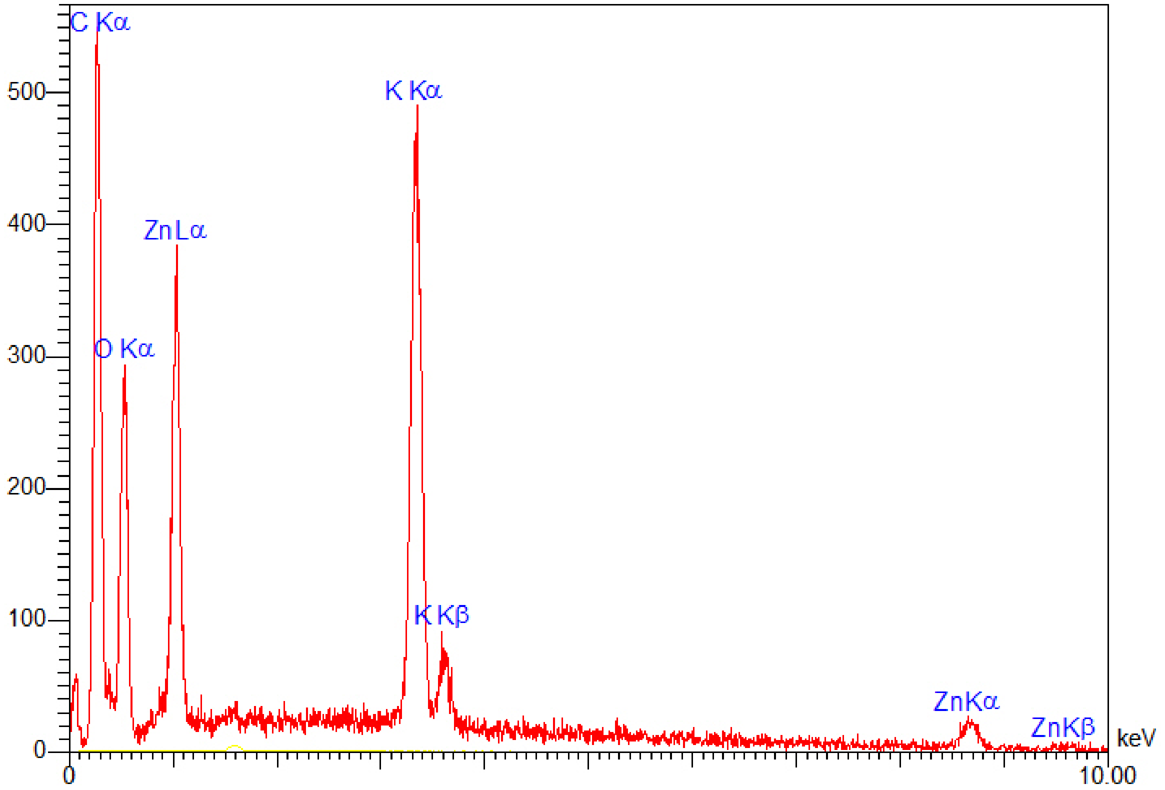

Figure 4S. EDX analysis of ZnQ_2**.**

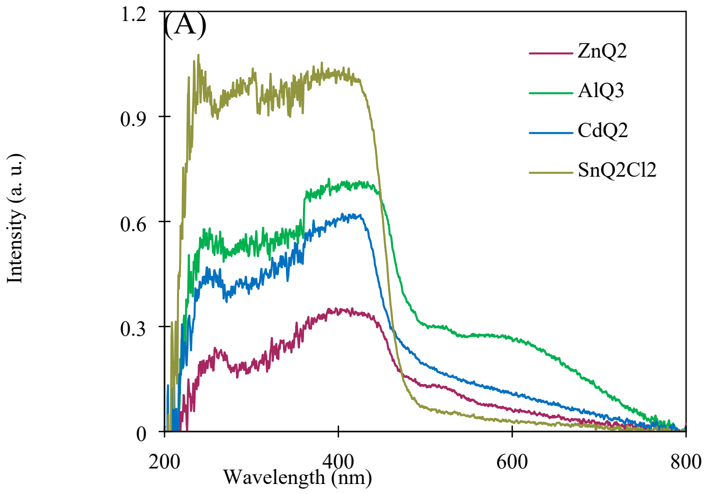

Figure 5S. DRS analysis of AlQ_3, ZnQ_2, CdQ_2 and SnQ_2Cl_2 organometallic electron transfer materials.

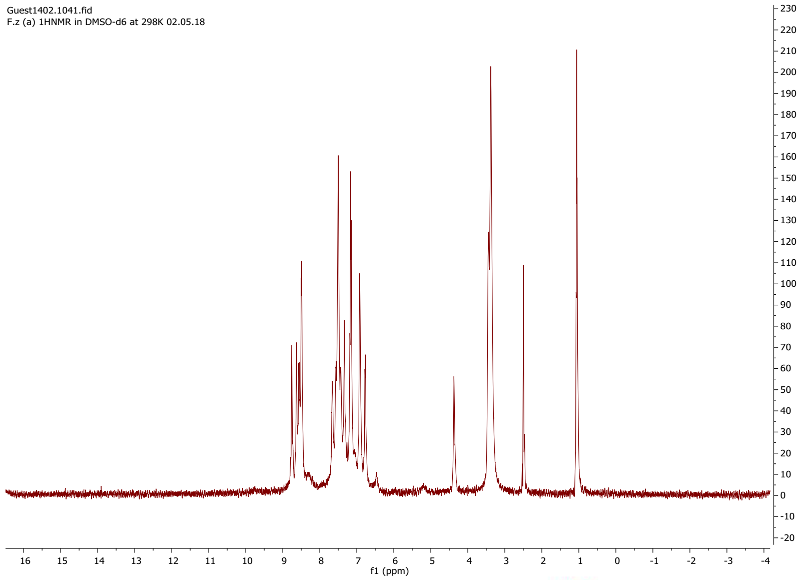
Guest1402.1041.fid
F.z (a) 1HNMR in DMSO-d6 at 298K 02.05.18
f1 (ppm)

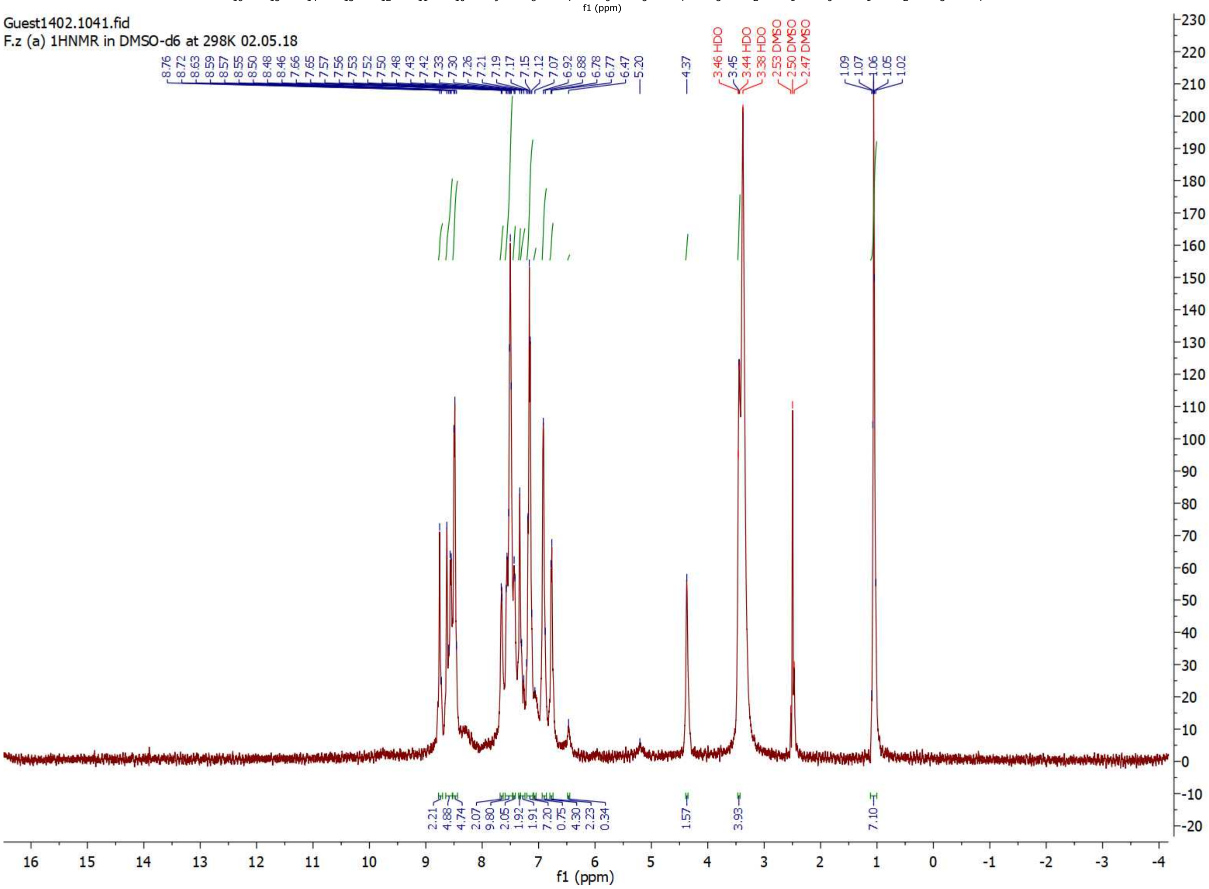
Guest1402.1041.fid
F.z (a) 1HNMR in DMSO-d6 at 298K 02.05.18
8.76 8.72 8.63 8.59 8.57 8.55 8.50 8.48 8.46 7.66 7.65 7.57 7.56 7.53 7.52 7.50 7.48 7.43 7.42 7.33 7.30 7.26 7.21 7.19 7.17 7.15 7.12 7.07 6.92 6.88 6.78 6.77 6.47 5.20
4.37
3.46 HDO 3.45 3.44 HDO 3.38 HDO 2.53 DMSO 2.50 DMSO 2.47 DMSO
1.09 1.07 1.06 1.05 1.02
2.21 4.88 4.74 2.07 9.80 2.05 1.92 1.91 7.20 0.75 4.30 2.23 0.34
1.57
3.93
7.10
f1 (ppm)

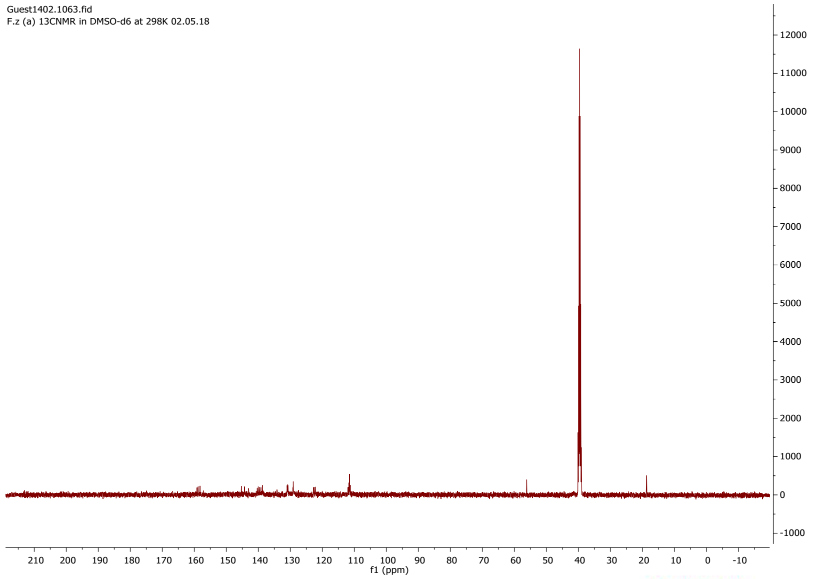
Guest1402.1063.fid
F.z (a) 13CNMR in DMSO-d6 at 298K 02.05.18
f1 (ppm)

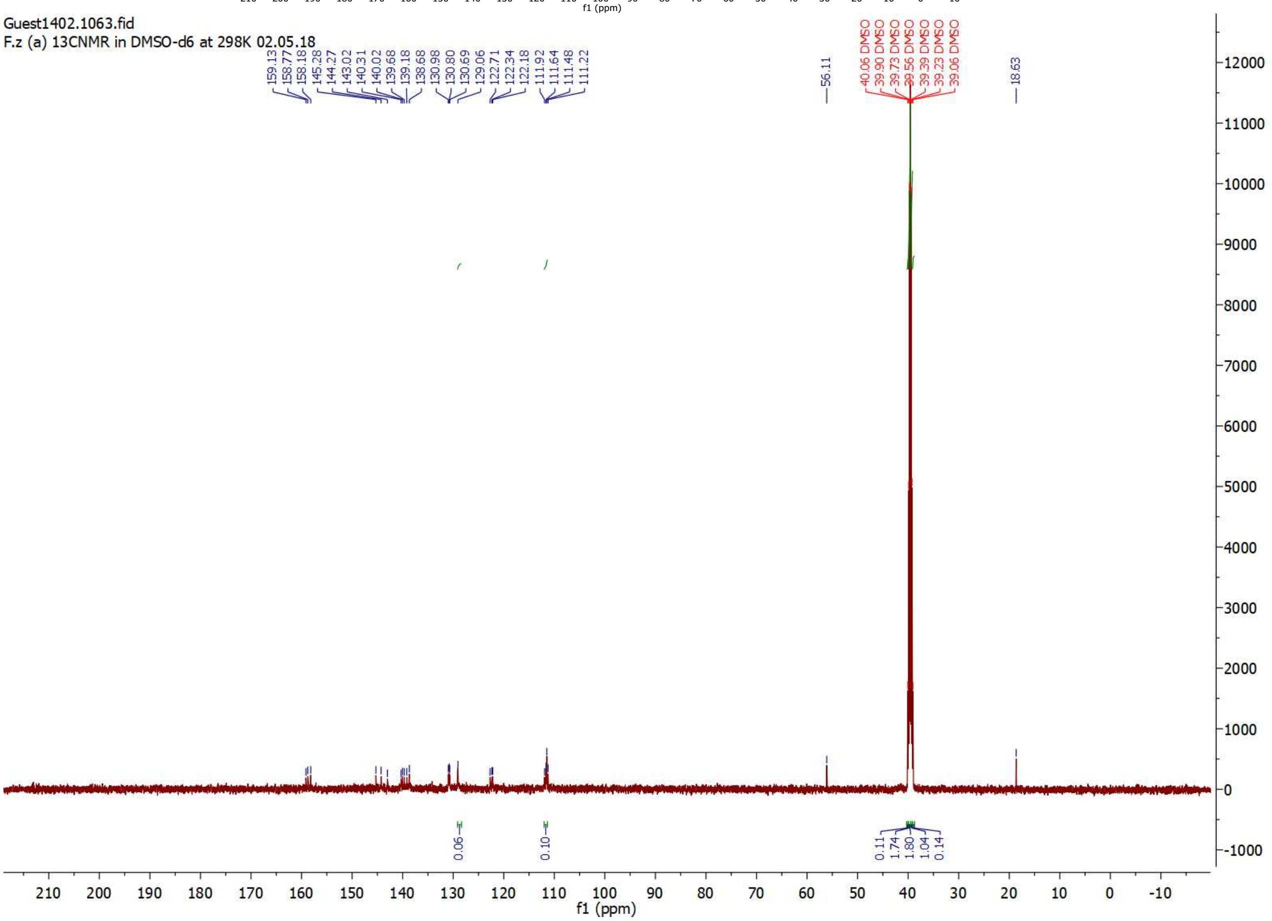
Guest1402.1063.fid
F.z (a) 13CNMR in DMSO-d6 at 298K 02.05.18
159.13
158.77
158.18
145.28
144.27
143.02
140.31
140.02
139.68
139.18
138.68
130.98
130.80
130.69
129.06
122.71
122.34
122.18
111.92
111.64
111.48
111.22
56.11
40.06 DMSO
39.90 DMSO
39.73 DMSO
39.56 DMSO
39.39 DMSO
39.23 DMSO
39.06 DMSO
18.63
0.06
0.10
0.11
1.74
1.80
1.04
0.14
f1 (ppm)

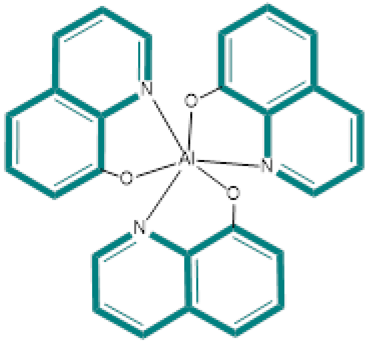

Figure 6S. ^{1}H & ^{13}C NMR analysis of AlQ_3.

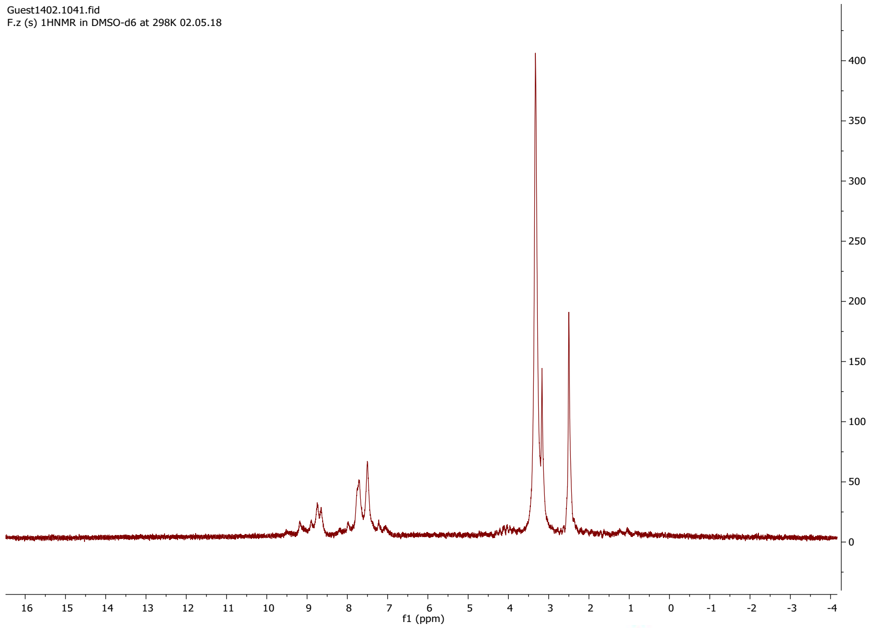

Guest1402.1041.fid
F.z (s) 1HNMR in DMSO-d6 at 298K 02.05.18
f1 (ppm)

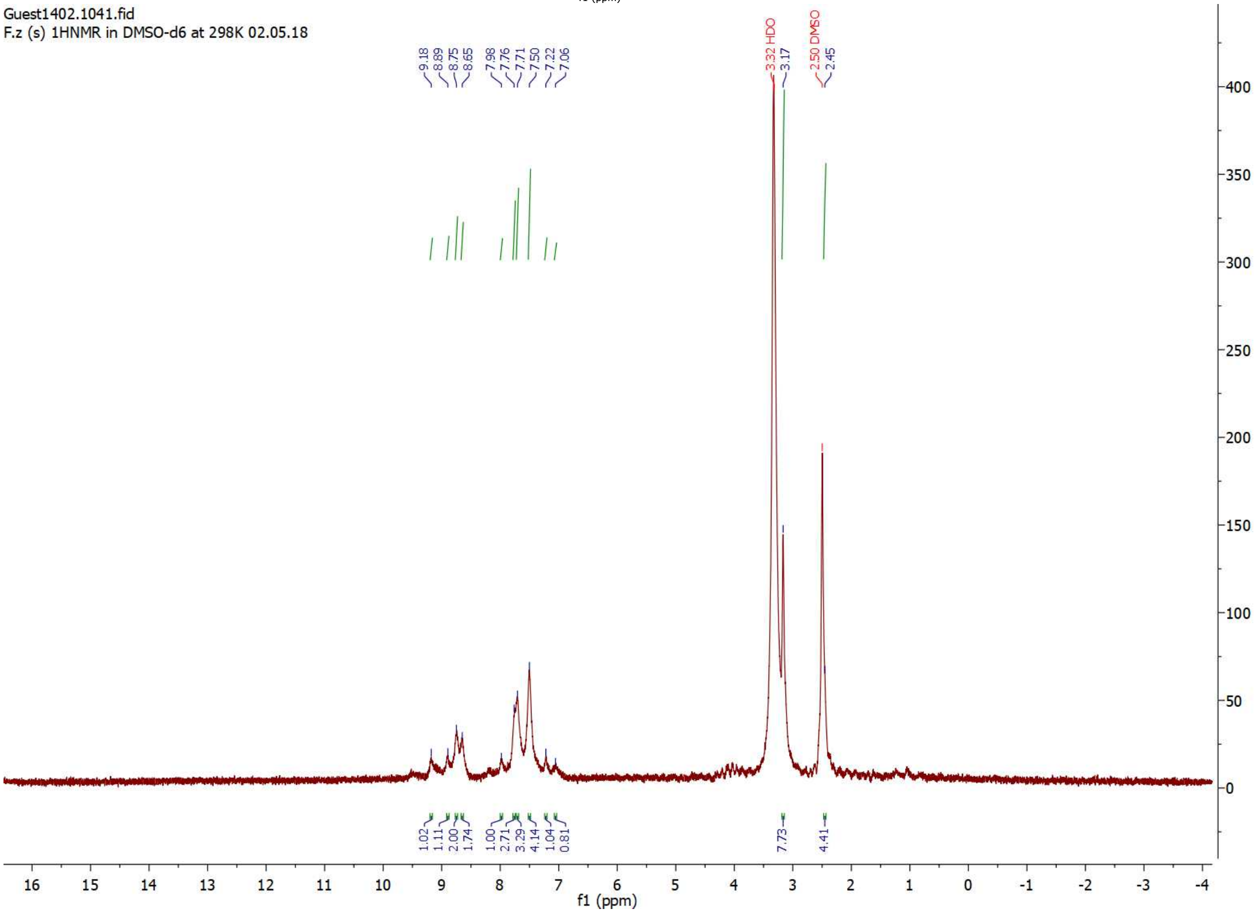

Guest1402.1041.fid
F.z (s) 1HNMR in DMSO-d6 at 298K 02.05.18
9.18
8.89
8.75
8.65
7.98
7.76
7.71
7.50
7.22
7.06
3.32 HDO
3.17
2.50 DMSO
2.45
1.02
1.11
2.00
1.74
1.00
2.71
3.29
4.14
1.04
0.81
7.73
4.41
f1 (ppm)

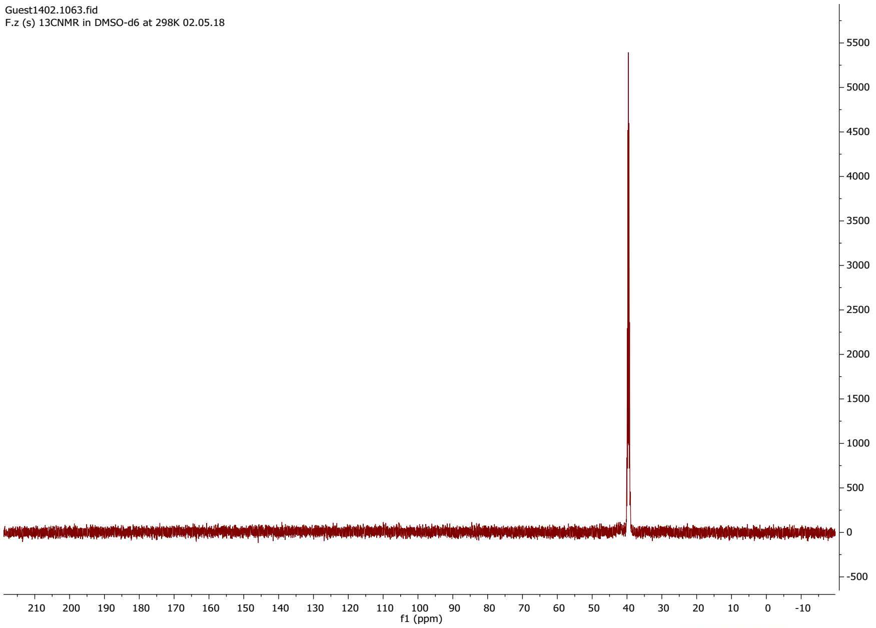
Guest1402.1063.fid
F.z (s) 13CNMR in DMSO-d6 at 298K 02.05.18
f1 (ppm)

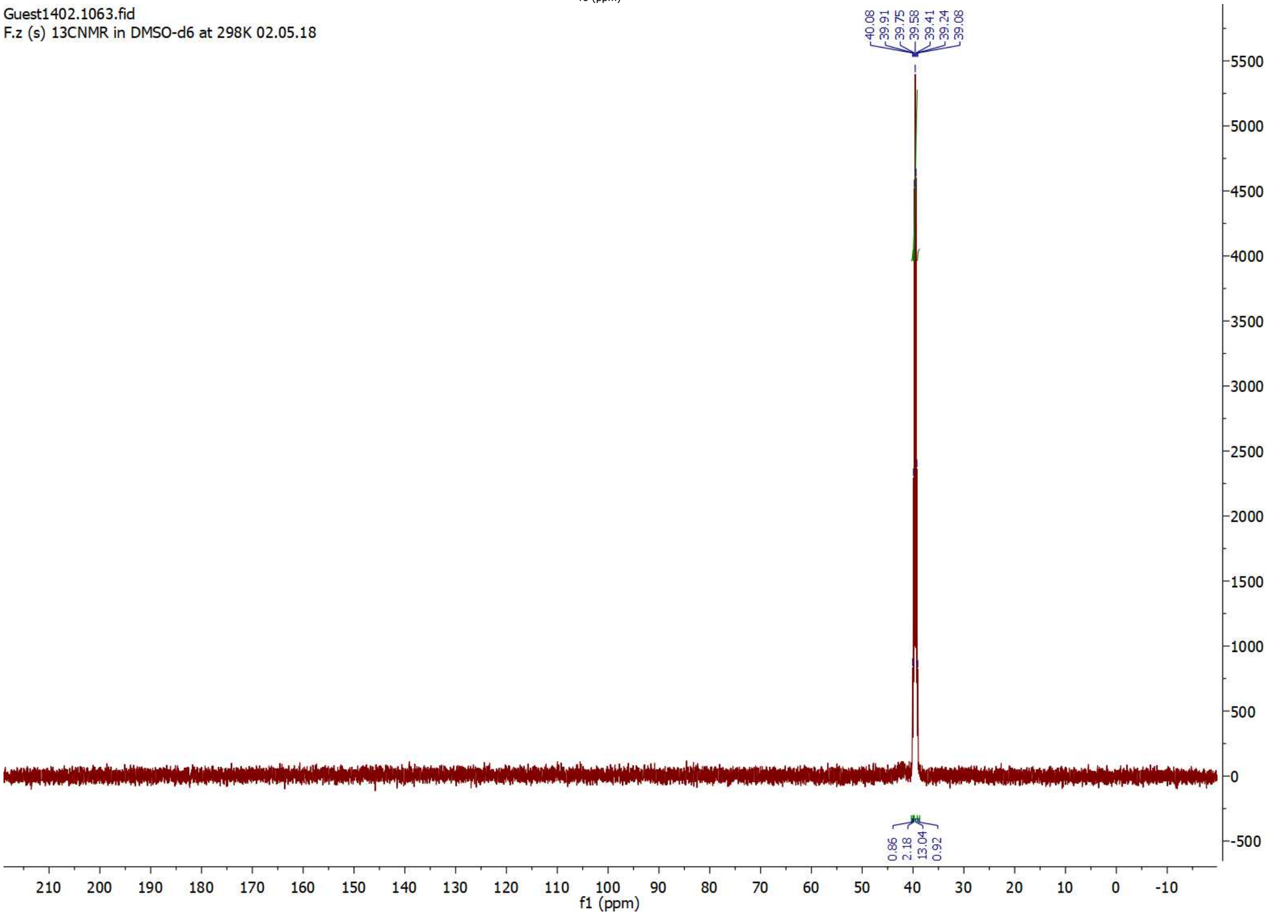
Guest1402.1063.fid
F.z (s) 13CNMR in DMSO-d6 at 298K 02.05.18
40.08
39.91
39.75
39.58
39.41
39.24
39.08
0.86
2.18
13.04
0.92
f1 (ppm)

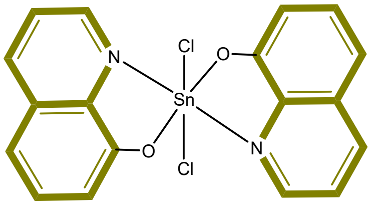

Figure 7S. ^{1}H & ^{13}C NMR analysis of SnQ_2Cl_2.

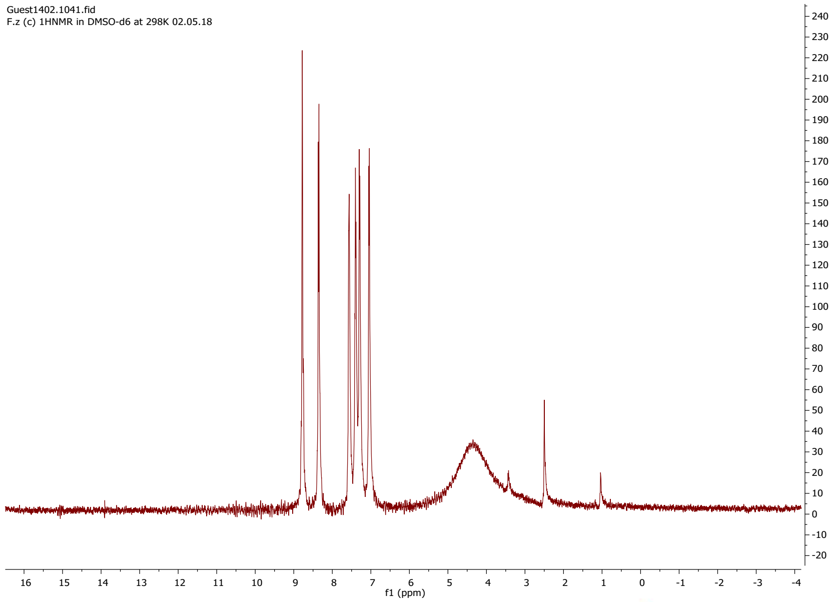
Guest1402.1041.fid
F.z (c) 1HNMR in DMSO-d6 at 298K 02.05.18
f1 (ppm)

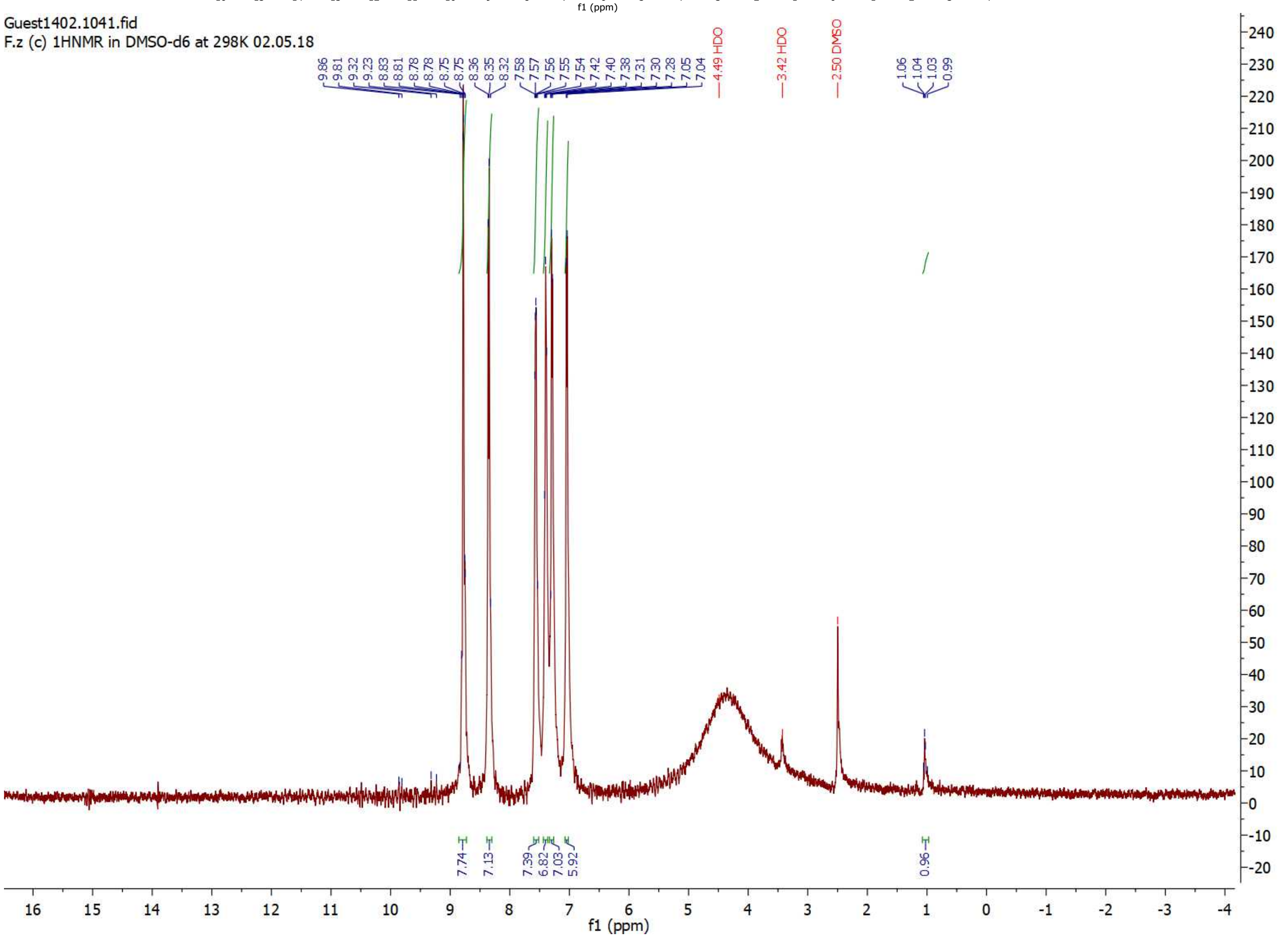
Guest1402.1041.fid
F.z (c) 1HNMR in DMSO-d6 at 298K 02.05.18
9.86
9.81
9.32
9.23
8.83
8.81
8.78
8.78
8.75
8.75
8.36
8.35
8.32
7.58
7.57
7.56
7.55
7.54
7.42
7.40
7.38
7.31
7.30
7.28
7.05
7.04
4.49 HDO
3.42 HDO
2.50 DMSO
1.06
1.04
1.03
0.99
7.74
7.13
7.39
6.82
7.03
5.92
0.96
f1 (ppm)

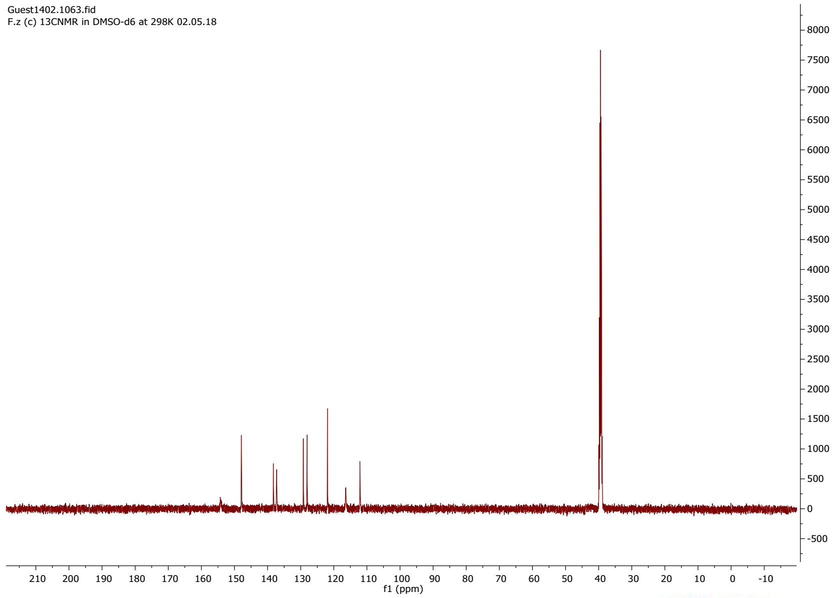
Guest1402.1063.fid
F.z (c) 13CNMR in DMSO-d6 at 298K 02.05.18
f1 (ppm)

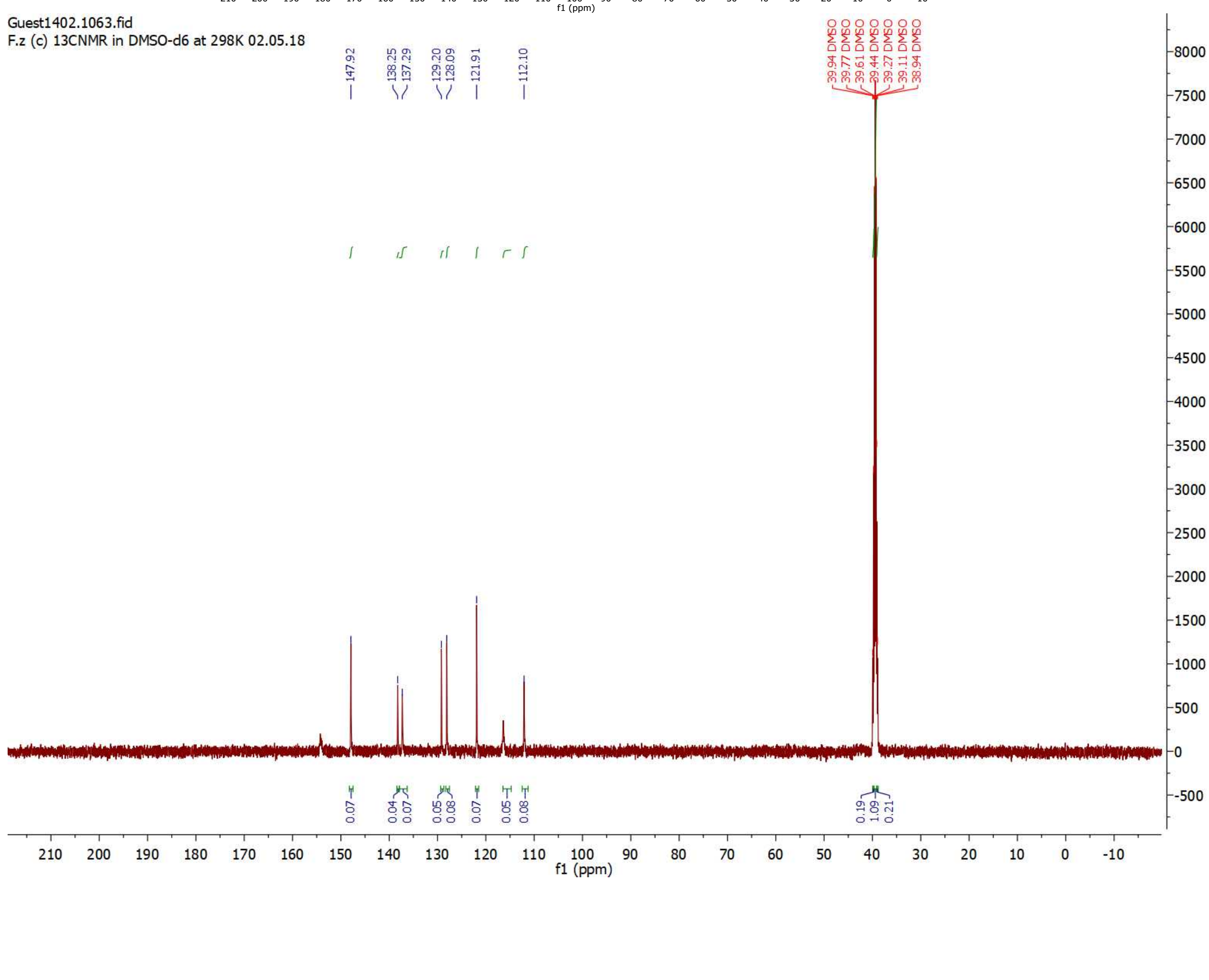
Guest1402.1063.fid
F.z (c) 13CNMR in DMSO-d6 at 298K 02.05.18
147.92
138.25
137.29
129.20
128.09
121.91
112.10
39.94 DMSO
39.77 DMSO
39.61 DMSO
39.44 DMSO
39.27 DMSO
39.11 DMSO
38.94 DMSO
0.07
0.04
0.07
0.05
0.08
0.07
0.05
0.08
0.19
1.09
0.21
f1 (ppm)

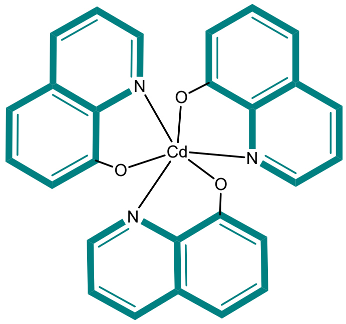

Figure 8S. ^{1}H & ^{13}C NMR analysis of CdQ_2.

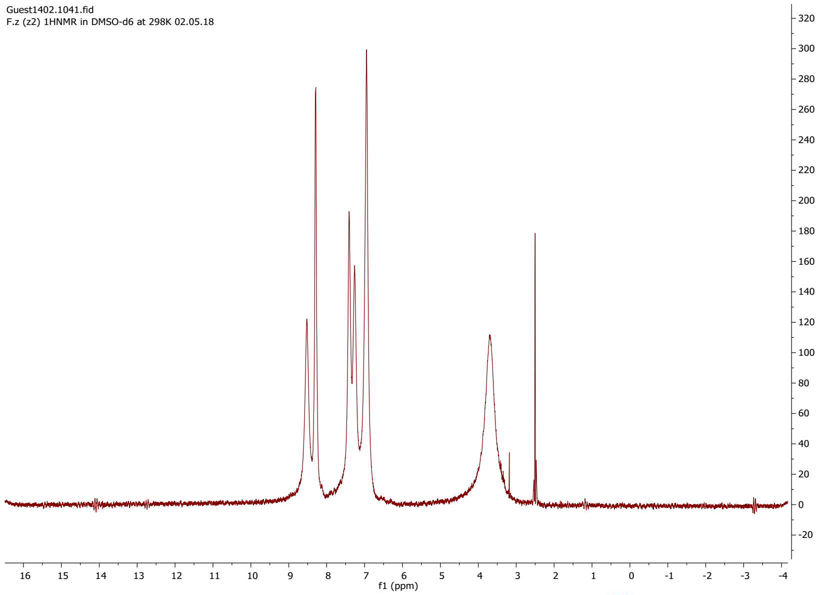
Guest1402.1041.fid
F.z (z2) 1HNMR in DMSO-d6 at 298K 02.05.18
f1 (ppm)

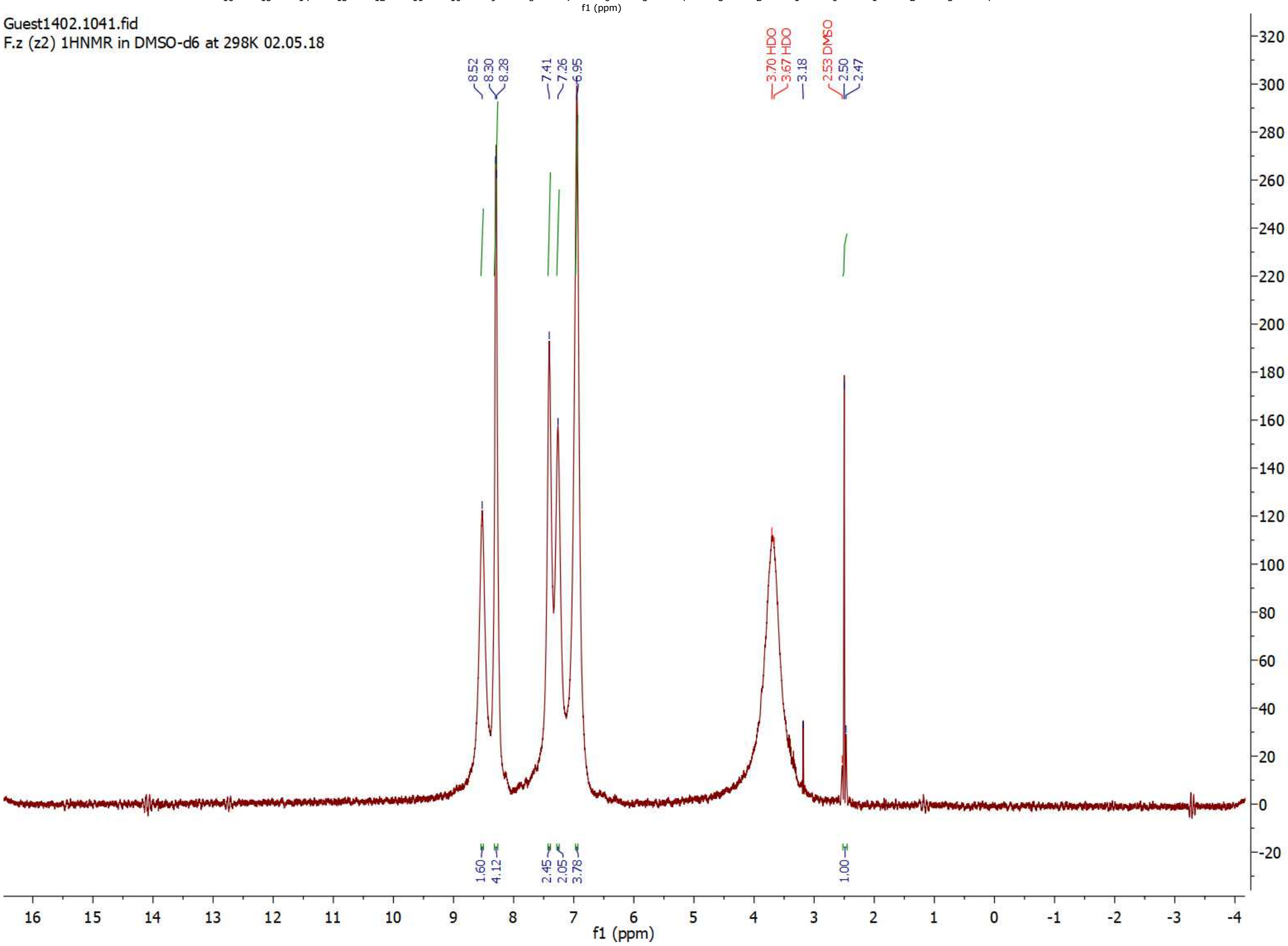
Guest1402.1041.fid
F.z (z2) 1HNMR in DMSO-d6 at 298K 02.05.18
8.52
8.30
8.28
7.41
7.26
6.95
3.70 HDO
3.67 HDO
3.18
2.53 DMSO
2.50
2.47
1.60
4.12
2.45
2.05
3.78
1.00
f1 (ppm)

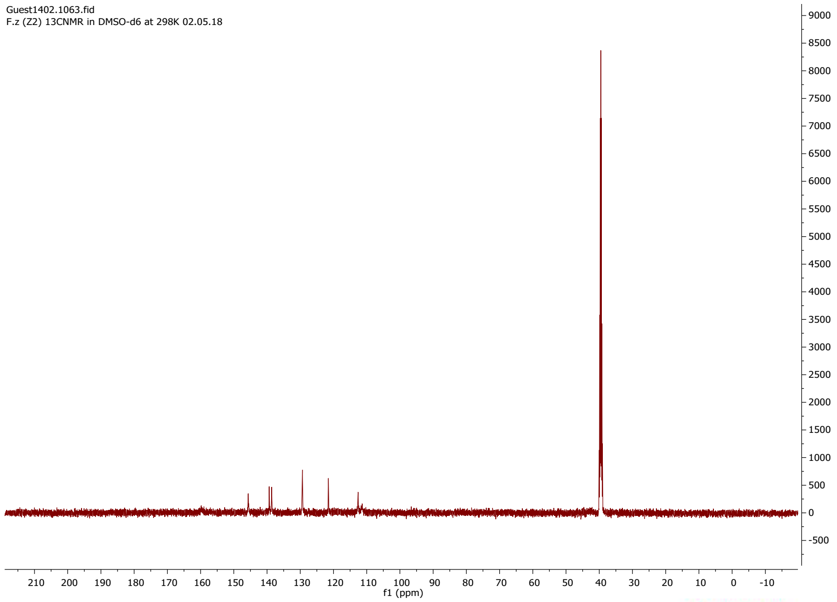

Guest1402.1063.fid
F.z (Z2) 13CNMR in DMSO-d6 at 298K 02.05.18
f1 (ppm)

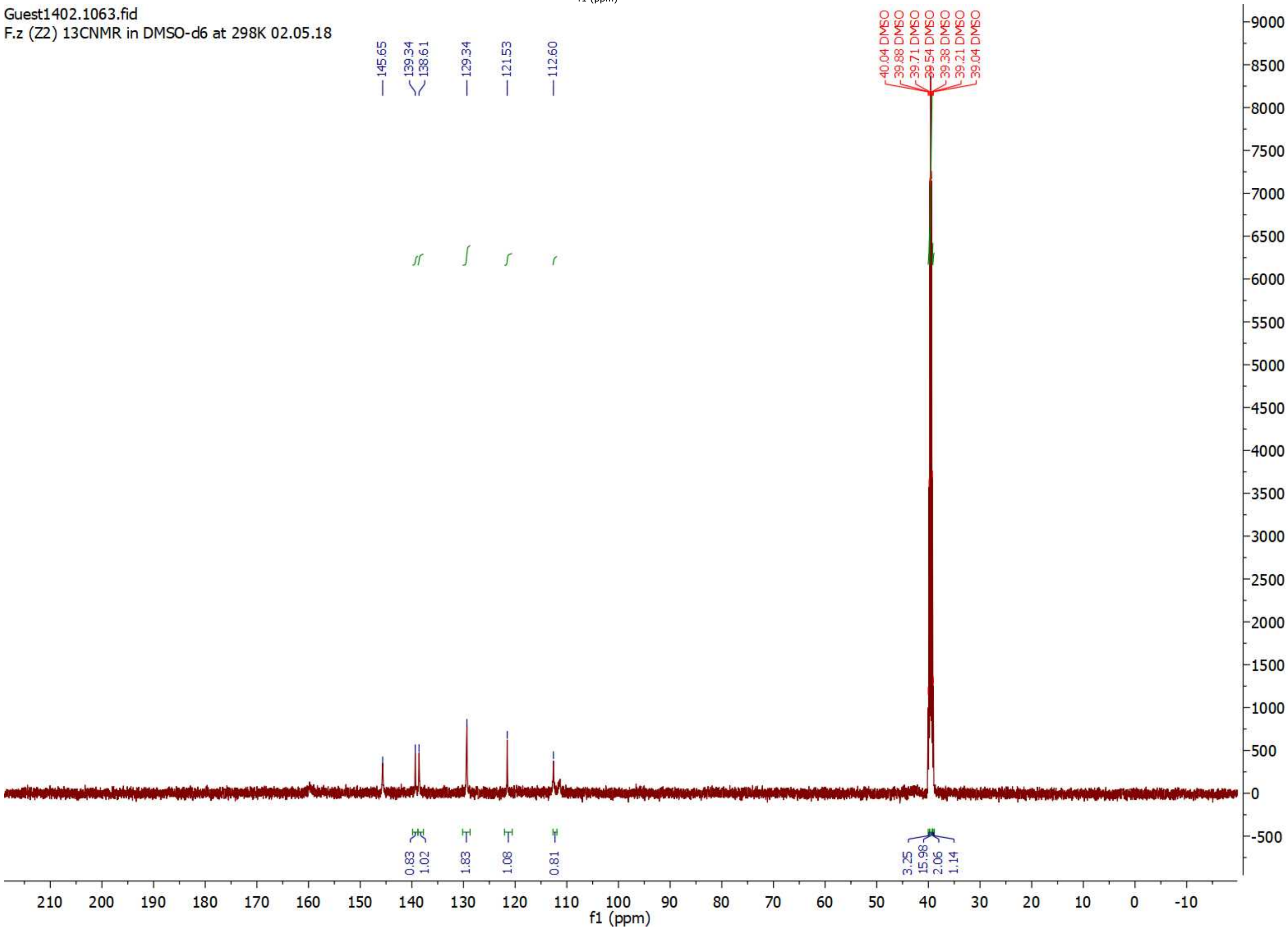

Guest1402.1063.fid
F.z (Z2) 13CNMR in DMSO-d6 at 298K 02.05.18
145.65
139.34
138.61
129.34
121.53
112.60
40.04 DMSO
39.88 DMSO
39.71 DMSO
39.54 DMSO
39.38 DMSO
39.21 DMSO
39.04 DMSO
0.83
1.02
1.83
1.08
0.81
3.25
15.98
2.06
1.14
f1 (ppm)

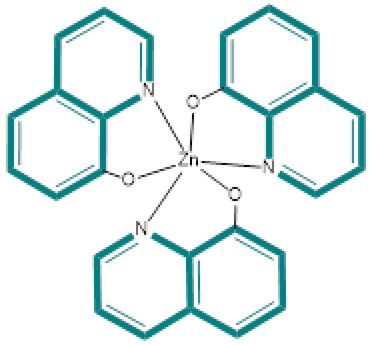

Figure 9S. ^{1}H & ^{13}C NMR analysis of ZnQ_2.
